# Phylogenetic congruence between subtropical trees and their associated fungi

**DOI:** 10.1002/ece3.2503

**Published:** 2016-10-26

**Authors:** Xubing Liu, Minxia Liang, Rampal S. Etienne, Gregory S. Gilbert, Shixiao Yu

**Affiliations:** ^1^ Department of EcologySchool of Life Sciences/State Key Laboratory of BiocontrolSun Yat‐sen UniversityGuangzhouChina; ^2^ Groningen Institute for Evolutionary Life SciencesUniversity of GroningenGroningenThe Netherlands; ^3^ Department of Environmental StudiesUniversity of California Santa CruzSanta CruzCAUSA

**Keywords:** congruent phylogenies, DNA sequencing, foliar pathogens, fungi, molecular phylogeny, network, soil pathogens, subtropical trees

## Abstract

Recent studies have detected phylogenetic signals in pathogen–host networks for both soil‐borne and leaf‐infecting fungi, suggesting that pathogenic fungi may track or coevolve with their preferred hosts. However, a phylogenetically concordant relationship between multiple hosts and multiple fungi in has rarely been investigated. Using next‐generation high‐throughput DNA sequencing techniques, we analyzed fungal taxa associated with diseased leaves, rotten seeds, and infected seedlings of subtropical trees. We compared the topologies of the phylogenetic trees of the soil and foliar fungi based on the internal transcribed spacer (ITS) region with the phylogeny of host tree species based on *matK*,* rbcL*,* atpB,* and *5.8S* genes. We identified 37 foliar and 103 soil pathogenic fungi belonging to the Ascomycota and Basidiomycota phyla and detected significantly nonrandom host–fungus combinations, which clustered on both the fungus phylogeny and the host phylogeny. The explicit evidence of congruent phylogenies between tree hosts and their potential fungal pathogens suggests either diffuse coevolution among the plant–fungal interaction networks or that the distribution of fungal species tracked spatially associated hosts with phylogenetically conserved traits and habitat preferences. Phylogenetic conservatism in plant–fungal interactions within a local community promotes host and parasite specificity, which is integral to the important role of fungi in promoting species coexistence and maintaining biodiversity of forest communities.

## 1 Introduction

Ecologists have proposed many mechanisms to explain the extraordinarily high diversity in the tropics. Plant–natural enemy feedback mechanisms, such as the Janzen–Connell hypothesis (Connell, 1971; Janzen, 1970), have been widely confirmed to play important roles in shaping the structure and dynamics of natural communities (Freckleton & Lewis, 2006). For these mechanisms to help maintain species diversity, local host specificity or selectivity of the enemies for host species is required, and as such, knowledge of the host range of pathogens is fundamental to understanding their impacts. However, most studies of pathogens in such feedback systems have either focused on specific plant–pathogen interactions (e.g., Gilbert, Hubbell, & Foster, 1994; Packer & Clay, 2000) or have treated the whole pathogen community as a black box without identifying its composition and diversity (e.g., Bever, 1994; Klironomos, 2002; Liu et al., 2012; Mangan et al., 2010; Petermann, Fergus, Turnbull, & Schmid, 2008; Spear, Coley, & Kursar, 2015). Neither approach provides a clear picture of how diverse sets of interacting host and pathogen species shape community diversity.

The high diversity of fungi living in plant tissue and soils (Hawksworth, 2012) provides a large pool of potential enemies, but many plant‐associated fungi can infect multiple hosts and individual plant hosts can be coinfected by multiple fungi (Barrett, Kniskern, Bodenhausen, Zhang, & Bergelson, 2009; Gilbert & Webb, 2007; Hersh, Vilgalys, & Clark, 2012). Multihost fungi vary in host specificity and ecological function depending on both host and environmental condition (Hersh et al., 2012), which makes it difficult to evaluate the impacts of many possible combinations of fungi. Hence, host performance measures, including survival, growth, and reproduction, are commonly used as integrated measures of functional host specificity for the whole fungal community and to represent outcomes of complex plant–fungal interactions (Bever, 1994; Klironomos, 2002; Liu et al., 2012; Mangan et al., 2010). However, direct measurement of the network of interactions among multiple hosts and multiple pathogens in natural communities is still in its infancy.

Recent studies found that the host ranges of multihost fungi are phylogenetically constrained—that is, the probability that a pathogenic fungus can infect two different plant species decreases continuously with the increase in phylogenetic distance between plant species (Gilbert & Webb, 2007; Liu et al., 2012). In our previous studies, we evaluated the negative plant–soil feedbacks mediated by soil biota, and documented a gradual increase in seedling survival with phylogenetic distance between tree species (Liu et al., 2012) as well as genetic distance among conspecific individuals (Liu, Etienne, Liang, Wang, & Yu, 2015). All these studies suggest phylogenetically conservative feedback between hosts and plant‐associated fungi, but the nonrandom association has not been measured directly nor examined in the framework of a network of interacting sets of plant and pathogen species.

Whereas networks of multiple pathogens with multiple hosts dominate natural systems (Barrett et al., 2009; Vacher, Piou, & Desprez‐Loustau, 2008), most previous work has emphasized single‐pathogen infections in a single plant species. Some recent studies have provided a window into the structure of bipartite networks of belowground plant–fungal interactions dominated by mycorrhizal fungi (e.g., Montesinos‐Navarro, Segarra‐Moragues, Valiente‐Banuet, & Verdú, 2012; Taylor et al., 2014; Toju, Guimarães, Olesen, & Thompson, 2014; Toju, Sato, et al., 2013, Toju, Yamamoto, et al., 2013), but such networks have included <2% putative pathogens. Those studies of mutualist plant–fungal networks, however, suggest plant–fungal networks may show stronger compartmentalization, lower interaction specialization, and less nestedness than is commonly associated with other mutualist networks.

In this study, we identified fungal taxa associated with diseased leaves, rotten seeds, and infected seedlings of subtropical trees, using next‐generation high‐throughput DNA sequencing techniques, to investigate how fungal and host species associate with each other given the locally available species pools. We focused mainly on the two dominant taxonomic groups of fungi, the Ascomycota and Basidiomycota, in the Dikarya (Kirk, Cannon, Minter, & Stalpers, 2008). In total, these two groups represent 79% of the described species of true fungi, and most plant pathogens are ascomycetes and basidiomycetes (Blackwell, 2011). Although the host–parasite associations for both foliar and soil pathogens proved to be phylogenetically conserved (Gilbert & Webb, 2007; Liu et al., 2012), the fungi associated with the soil and foliar samples are expected to be very different because of different dispersal capacities and habitat requirements. We used different sequencing technologies for the two groups and analyzed the soil and foliar pathogenic fungal networks separately. We compared the topology of phylogenetic trees of the soil and foliar pathogenic fungi to the host tree phylogeny, to analyze the structure and specificity of the multiple fungi–multiple host interaction network of plant–pathogen associations in natural communities. Because most pathogens can attack multiple hosts but their host ranges are constrained by phylogenetically conserved traits that reflect evolutionary relationships important in plant–fungal interactions, we expect to find closely related hosts linked by shared pathogens and closely related pathogens using similar sets of host species.

## 2 Materials and Methods

### 2.1 Study site and selected species

We conducted fieldwork at Heishiding Nature Reserve (111°53′E, 23°27′N, 150–927 m above sea level), Guangdong Province, in south China. The reserve covers approximately 4,200 ha of subtropical evergreen broad‐leaved forest and has a subtropical moist monsoon climate. The mean annual temperature is 19.6°C with the lowest mean monthly temperature of 10.6°C in January and the highest of 28.4°C in July. Annual precipitation is 1,744 mm on average, occurring mainly between April and September (79% of annual rainfall), with a pronounced dry season from October to March.

We chose 26 evergreen broad‐leaved tree species that commonly occur in the study area (Table 1). For each of the 26 species, we searched GenBank (Benson et al., 2013) for four gene sequences: *matK*,* rbcL*,* atpB,* and *5.8S*, which are the standardized DNA barcodes for land plants (CBOL Plant Working Group 2009) and are commonly used in published angiosperm phylogenies (e.g., Cadotte, Cardinale, & Oakley, 2008; Wojciechowski, Lavin, & Sanderson, 2004). Of the 26 species, 24 had at least one gene represented in GenBank. For each of the two remaining species, *Quercus chungii* and *Ormosia pachycarpa*, we used gene sequences of congeneric relatives (Cadotte et al., 2008), which were *Quercus myrsinifolia* and *Ormosia fordiana*, respectively. The GenBank accession numbers for all the sequences used are shown in Table 1.

**Table 1:** The list of focal tree species, GenBank sequence accession numbers, and whether soil or foliar fungal associates were sequenced

Species	Family	Order	GenBank accession number				Soil fungi	Foliar fungi
			*matK*	*rbcL*	*atpB*	*5.8S*		
*Diospyros morrisiana*	Ebenaceae	Ericales	HQ427383	HQ427240	NA	NA	√	
*Ardisia quinquegona*	Myrsinaceae	Ericales	HQ415400	GQ436753	NA	FJ980441	√	
*Symplocos adenophylla*	Symplocaceae	Ericales	AY336364	NA	NA	AY336308	√	√
*Symplocos laurina*	Symplocaceae	Ericales	AY336369	NA	NA	AY336317	√	
*Schima superba*	Theaceae	Ericales	HQ415305	AF421103	AF420982	HM100443	√	
*Ormosia glaberrima*	Fabaceae	Fabales	HQ415279	HQ415097	NA	NA	√	√
*Ormosia pachycarpa*	Fabaceae	Fabales	HQ415278	HQ415096	NA	NA		√
*Castanopsis carlesii*	Fagaceae	Fagales	JF953446	JF941153	FJ185060	AY040372	√	
*Castanopsis fabri*	Fagaceae	Fagales	EF057132	JF941169	NA	NA	√	√
*Castanopsis fissa*	Fagaceae	Fagales	EF057128	JF941179	NA	AY040391	√	
*Quercus chungii*	Fagaceae	Fagales	AB060063	AB060572	FJ185068	AF098414		√
*Engelhardia fenzelii*	Juglandaceae	Fagales	AY147099	AY147095	AY263951	NA	√	
*Cinnamomum parthenoxylon*	Lauraceae	Laurales	GQ434288	JX843239	NA	NA		√
*Cinnamomum pauciflorum*	Lauraceae	Laurales	HM019323	HM019463	NA	NA		√
*Cryptocarya concinna*	Lauraceae	Laurales	HQ415284	HQ415104	NA	NA	√	
*Lindera chunii*	Lauraceae	Laurales	HM019334	HM019474	NA	DQ124266	√	√
*Litsea acutivena*	Lauraceae	Laurales	NA	NA	NA	DQ120605		√
*Litsea elongata*	Lauraceae	Laurales	HQ427403	HQ427261	NA	DQ120606	√	√
*Machilus breviflora*	Lauraceae	Laurales	JF954532	JF942446	NA	FJ755434	√	
*Neolitsea phanerophlebia*	Lauraceae	Laurales	JF954720	JF942627	NA	JF977154	√	√
*Manglietia moto*	Magnoliaceae	Magnoliales	AF123477	NA	NA	NA		√
*Elaeocarpus sylvestris*	Elaeocarpaceae	Oxalidales	HQ415265	HQ415081	AB111774	NA	√	
*Artocarpus styracifolius*	Moraceae	Rosales	HQ415243	HQ415055	NA	NA	√	
*Canarium album*	Burseraceae	Sapindales	HQ415266	FJ466626	NA	NA	√	√
*Altingia chinensis*	Hamamelidaceae	Saxifragales	AF133225	DQ352376	EU595847	AF162219	√	
*Itea chinensis*	Saxifragaceae	Saxifragales	HQ415356	HQ415186	NA	NA	√	

For each species, we randomly selected five adult trees that had conspecific rotten seeds or infected seedlings at a distance of 0–2 m. All the sampled individuals were located within an area of 20 ha in the field, while conspecific individuals were at least 100 m apart and widely distributed across the sampling area (see Figure S1 for the spatial sampling map), to make sure they were not spatially autocorrelated.

### 2.2 Pathogen isolation from the soil and DNA sequencing

To assess the potential association of soil fungi with host trees, we selected five independent adult trees for each focal species, each of which had rotten seeds and infected seedlings with damping‐off diseases within 0–2 m. For each tree, one 5‐g sample of soil (0–10 cm deep) was then collected around a single diseased seed or seedling in late August 2012 (the end of the wet season). We treated the rotten seeds and infected seedlings as indicators of pathogen emergence and assumed that fungal communities in these soil samples were good representatives of pathogens associated with corresponding adult trees and caused disease on seeds and seedlings. We extracted total genomic DNA directly from each sample according to a standard procedure. The nuclear ribosomal ITS region (including ITS1, 5.8S rRNA,and ITS2), an approximately 600‐base‐pair region frequently used in species‐level systematics for fungi, was chosen as the target gene because it has been proposed as the standard barcode for fungi and has the highest probability of successful identification for the broadest range of fungi, with the most clearly defined barcode gap between inter‐ and intraspecific variations (Schoch et al., 2012). The ITS gene was amplified by polymerase chain reactions (PCRs) using the fungal primer set of ITS1 (5′‐*NNNNNNNNNN*TCCGTAGGTGAACCTGCGG‐3′) and ITS4 (5′‐TCCTCCGCTTATTGATATGC‐3′) (White, Bruns, Lee, & Taylor, 1990), where *NNNNNNNNNN* represents the 10‐bp barcode designed for sample identification and the ITS primers were fused with the 454 pyrosequencing adapters. The PCRs were carried out in a 20‐μl reaction mixture containing 4 μl 5× FastPfu Buffer, 0.4 μl FastPfu polymerase, 2 μl 2.5 mmol/L dNTP mix (all from Beijing TransGen Biotech Co., Ltd., Beijing, China), 0.8 μl 5 μmol/L of each primer, and 10 ng of template DNA. The PCR amplification conditions were as follows: initial denaturation at 95°C for 2 min, 35 cycles of denaturation at 95°C for 30 s, primer annealing at 50°C for 30 s, extension at 72°C for 30 s, and a final extension of 5 min at 72°C. For each soil sample, the PCR was conducted in triplicate and the PCR products were pooled to reduce PCR amplification biases. The products were then purified using an AxyPrep™ DNA gel extraction kit (Axygen Biosciences, CA, USA). The amplicon library was constructed by pooling approximately equal amounts of the amplification products from individual soil samples. Each sample was quantified and the appropriate volume of the cleaned PCR amplicons was combined as previously described (Fierer, Hamady, Lauber, & Knight, 2008), where the amplification products were normalized in equimolar amounts to produce equivalent sequencing depth from all samples. The composite DNA sample was sequenced using a Genome Sequencer 454 FLX System (Roche 454 Life Sciences, CT, USA) with two lanes used.

### 2.3 Pathogen isolation from leaves and DNA sequencing

**Figure 1:**
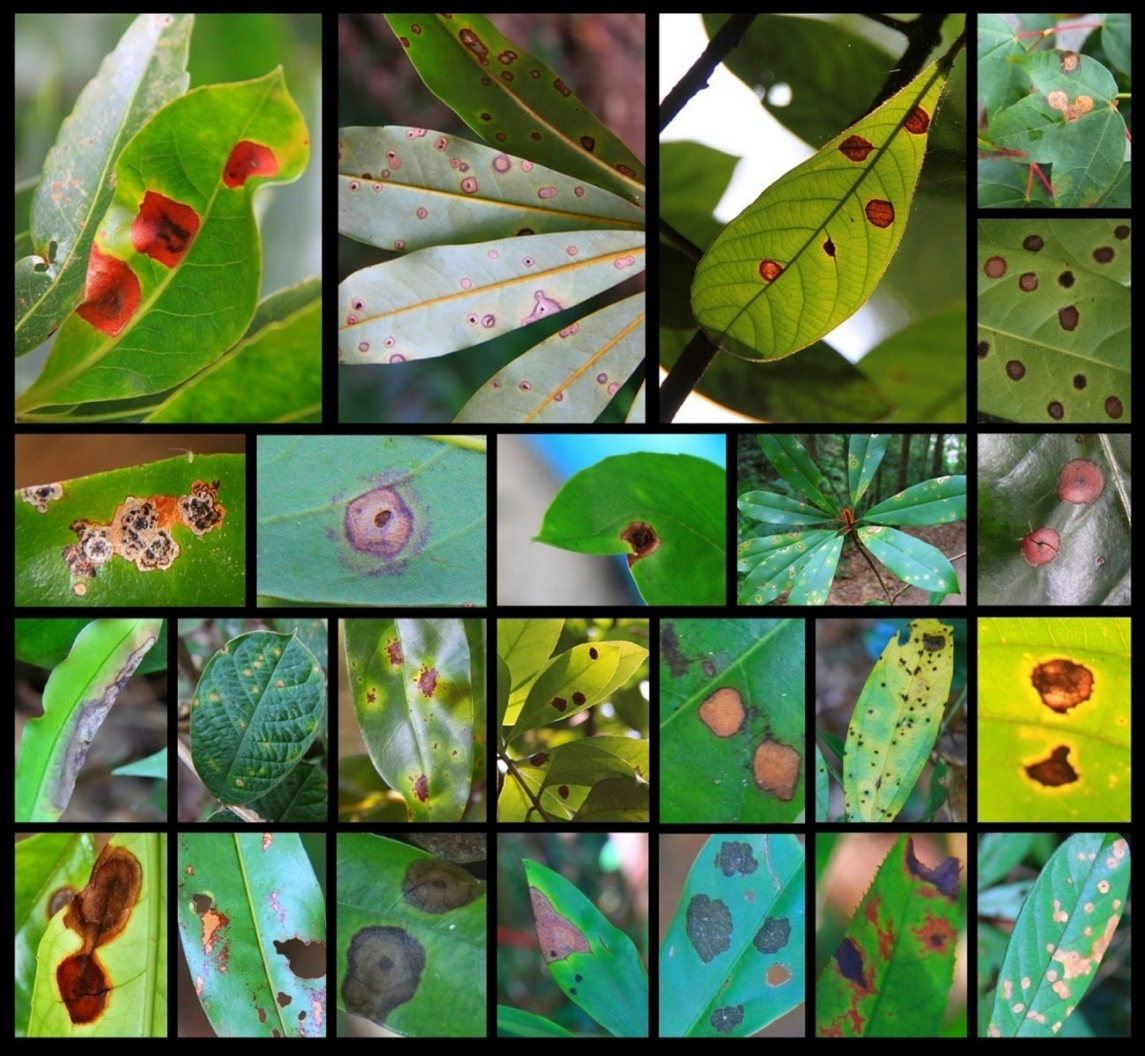
Examples of symptoms of fungal disease on leaves from which DNA sequences were obtained

For each focal species, we randomly chose five adult trees (Figure S1) and collected diseased leaves with obvious necrosis and chlorosis caused by fungal symbionts (Figure 1), placed them in plastic bags, and returned to the laboratory for processing within 2 hr. Six to 15 leaves from different branches were collected for each focal tree, depending on the range of fungal symbionts. We clipped out the infected area and cut it into small fragments (2 mm^2^) of tissue from the edge of the symptomatic area. Fragments were thoroughly mixed for each sampled adult, and then, exactly 10‐g fragments were surface‐sterilized by immersing them in 90% ethanol for 10 s, 10% commercial bleach (0.525% sodium hypochlorite) for 2 min, and then 70% ethanol for 2 min. Total genomic DNA was then extracted directly from each sample, and the fungal ITS rDNA genes were amplified with the ITS1 and ITS4 primers. The PCR products were purified using an AxyPrep™ DNA gel extraction kit (Axygen Biosciences, CA, USA) and then cloned using a TOPO‐TA cloning kit (Invitrogen, CA, USA). Hundred clones of each sample were directly sequenced. DNA extraction, PCR, and sequencing were all carried out by Invitrogen (Invitrogen Life Technologies, Shanghai, China). Because the total genomic DNA for each leaf sample was mainly composed of plant DNA with a small proportion fungal DNA, we used a different sequencing method and fewer leaf samples were successfully sequenced because of the lower fungal contents compared to soil samples.

### 2.4 Constructing the phylogenies

The analysis of sequence data and construction of phylogenies were conducted separately for leaf and soil fungi. We processed the sequence data using MOTHUR version 1.29 (Schloss et al., 2009). We first denoised the dataset as previously described (Schloss, Gevers, & Westcott, 2011). Using a Phred quality score of 20 as the threshold (predicted to have an accuracy of 99% or higher), we created the denoised dataset identifying and removing low‐quality reads that had ambiguous base calls, eight or more homopolymer bases, or average quality <25 bases. We then assigned the obtained high‐quality reads to each sample according to the 10‐bp barcodes and also removed the primer sequences of ITS1 and ITS4. We identified and removed chimeric sequences in each sample using UCHIME (Edgar, Haas, Clemente, Quince, & Knight, 2011), with the datasets themselves as the references. For the full datasets, we identified operational taxonomic units (OTUs) at the sequence identity level of 97% with the UCLUST algorithm (Edgar, 2010), and selected a representative sequence from each OTU for further alignment and phylogenetic analyses. For each OTU, the most abundant sequence was chosen as its representative sequence, while most of them were 450–530 bp in length. There was a relatively low length variation among aligned OTUs. For each host tree species, when an OTU of a soil or foliar fungus was found in three or more samples among the five samples, it was treated as an effective host–fungus combination (i.e., the corresponding fungal species of the OTU was regarded as a highly possible active invader of the host). The OTU sequences from soil or foliar samples were combined during this assignment to host species, but subsequent network analyses treated foliar and soil fungi separately.

For taxonomic assignment, we compared the OTU representative sequences against the UNITE ITS sequences database (Abarenkov et al., 2010), using BLAST (Altschul, Gish, Miller, Myers, & Lipman, 1990) with an expected *e*‐value of <10^−3^ and a minimum identity of 90%. Because it was not possible to reliably assign most OTUs to particular fungal species, each OTU was assigned to genus level. We subsequently investigated records of pathogenicity of each genus in the literature, and subsequent analyses only include OTUs belonging to genera of fungi that are commonly reported to contain many species of plant pathogens (Tables S1 and S2).

For DNA sequences from host plants (including *matK*,* rbcL*,* atpB,* and *5.8S*, which were combined into one matrix with unlinked partitions), as well as the ITS sequences of soil pathogens and foliar pathogens, we aligned sequences independently for each gene using MUSCLE in MEGA 5.2.2 (Tamura et al., 2011) with the default settings. The minimum length of the representative fungal OTUs was 434 bp, which means that all the aligned fungal sequences contained the full ITS region (including ITS1, 5.8S, and ITS2). While the ITS1 and ITS2 are relatively variable among taxa, the intercalary 5.8S gene is very conserved and can be aligned across the fungal phyla (Schoch et al., 2012). We selected best‐fit nucleotide substitution models for each gene using the Akaike Information Criterion as implemented in jModelTest 2.1.4 (Darriba, Taboada, Doallo, & Posada, 2012). We then used BEAST 1.8.0 (Drummond, Suchard, Xie, & Rambaut, 2012) to construct the Bayesian phylogenies with the aligned sequences, assuming relaxed uncorrelated lognormal clock models and all other parameters on default settings. Each Bayesian analysis was run for 10 million generations with a sampling frequency of 1,000 and a burn‐in of 10%. We examined stationarity and effective sample sizes (>200) using Tracer 1.5, and constructed majority rule consensus trees with mean node heights from the posterior distribution using TreeAnnotator 1.7.1 (Drummond et al., 2012). All the representative OTU sequences that were used for taxonomic assignment, alignment, and phylogeny construction in this study were uploaded to the European Nucleotide Archive database (accession numbers: LT547727–LT547800).

### 2.5 Statistical analyses

We used the permutation‐based ParaFit test for significant global association between “hosts” (subtropical trees) and “parasites” (foliar and soil fungi) (Legendre, Desdevises, & Bazin, 2002). Statistical assessment of a hypothesis of nonrandom host–parasite association requires a combination of three types of information: the phylogeny of the hosts, the phylogeny of the parasites, and the observed host–parasite associations based on the presence/absence of fungi. The null hypothesis (H_0_) is that emergence of the hosts and parasites has been independent (Legendre et al., 2002). The principal‐coordinate‐transformed phylogenetic distance data were analyzed with the “parafit” function in the R package “ape”, using 9,999 permutations.

Based on the qualitative 0/1 matrixes consisting of rows and columns represented plant species and fungal genera, respectively, we also determined whether the plant–fungus networks were statistically compartmentalized by conducting a modularity analysis using MODULAR 0.21 (Marquitti, Guimarães, Pires, & Bittencourt, 2014), with Barber's metric maximized and 999 randomizations for both null models. Modularity reflects the fact that there are groups of species that tend to interact more within species in the same group than is expected by chance.

## 3 Results

We successfully extracted and sequenced the fungal ITS rDNA genes with primers ITS1 and ITS4 from foliar samples of 13 species and soil samples of 20 species of the 26 focal tree species (Table 1). The Moran's *I* Autocorrelation Index weighted by the spatial distances among all the sampled individuals was not significantly different from that expected in a random distribution (Moran's *I *± *SD*: −0.020 ± 0.017, *p *=* *.469), indicating that the conspecific adult individuals were randomly distributed within the sampling area and not spatially clumped.

For the soil samples, we obtained 4883.5 ± 119.4 (mean ± *SE*) high‐quality sequences per sample, and 11.35% of them were identified as fungi with a mean length of 556 bp. For the leaf samples, we cloned and sequenced 96.2 ± 1.4 valid fungal ITS sequences for each sample, and the mean length of these genes was 570 bp. By comparing the OTU representative sequences against the UNITE ITS sequences database, we assigned each OTU to genus level for the 774 foliar fungal OTUs and the 1,387 soil fungal OTUs. We included only genera of fungi commonly reported to contain many species of pathogenic fungi (Tables S1 and S2). These fungi included classes Dothideomycetes, Sordariomycetes, Eurotiomycetes, Leotiomycetes, and Lecanoromycetes from the phylum of Ascomycota and occasional basidiomycete fungi from the Pucciniomycotina and Ustilaginomycotina.

**Figure 2:**
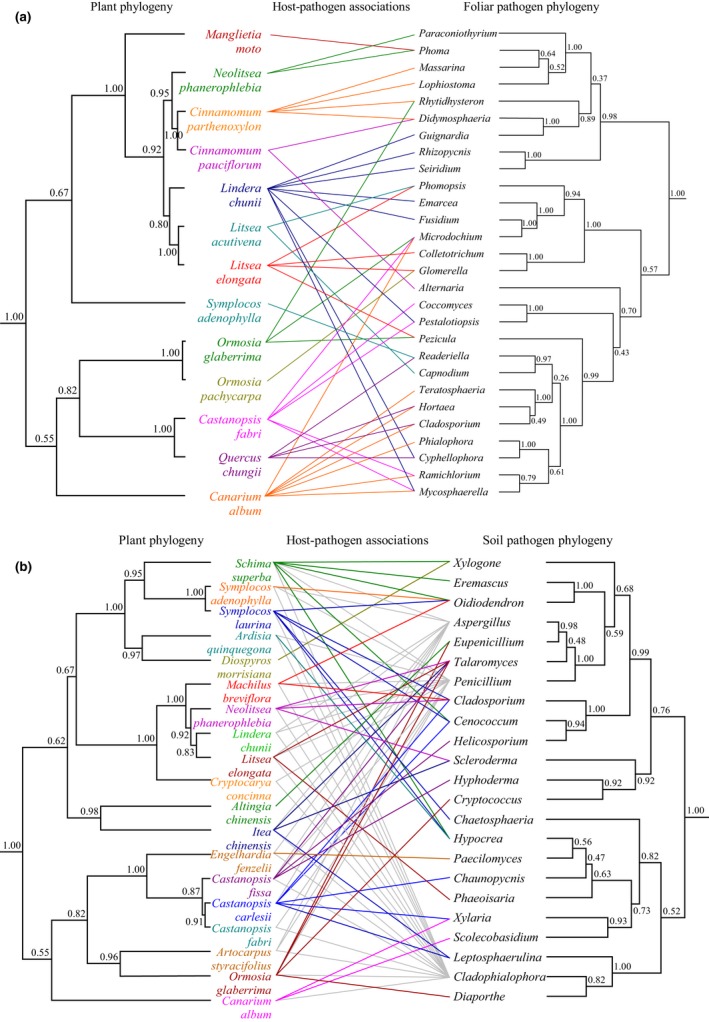
Phylogenetic trees of subtropical tree species and (a) leaf endophytic fungi or (b) soil‐borne fungi. The host phylogeny was inferred using Bayesian analysis of the combined, four‐gene dataset, including *matK*,* rbcL*,* atpB,* and *5.8S*. Operational taxonomic units (OTUs) of the nuclear ribosomal internal transcribed spacer region (ITS rDNA gene) of pathogenic fungi were compared against UNITE ITS sequences database using BLAST and assigned to genus level, and the fungus phylogenies were constructed using Bayesian analysis of the ITS gene (including ITS1, 5.8S rRNA, and ITS2). Numbers at nodes indicate Bayesian posterior probabilities. We considered a host and fungal pair to be associated when that fungal genus appeared on at least three of five individuals of that host species. For soil‐borne fungi, associations between hosts and three broad‐spectrum genera *Aspergillus*,* Penicillium,* and *Cladophialophora* were grayed to make them look less dominant

**Figure 3:**
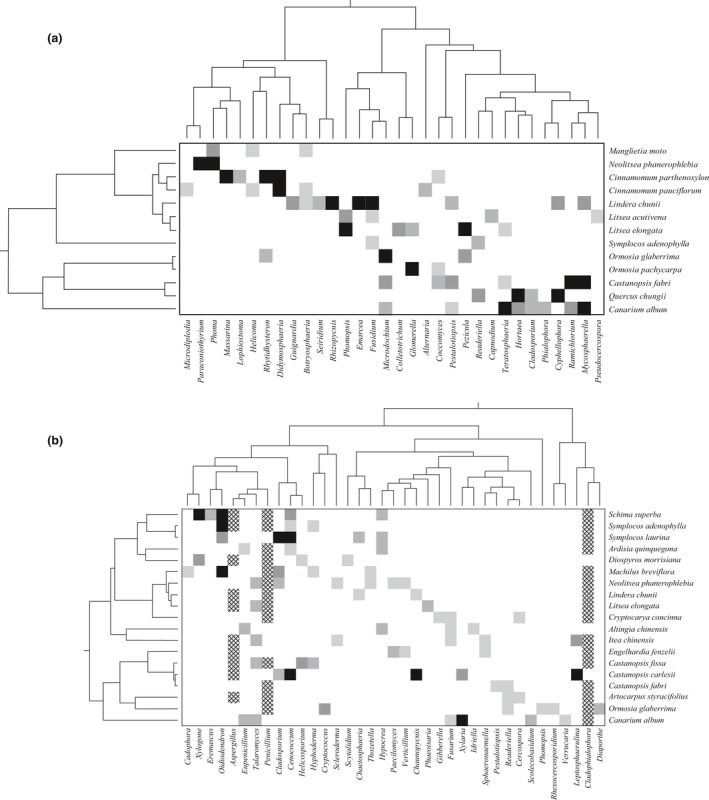
Schematic representation of host–fungi associations and the phylogenetic structure of subtropical tree species and (a) leaf endophytic fungi or (b) soil‐borne fungi. Each row and column depicts a plant and a fungal species, respectively, and plant and fungus phylogenies are shown besides and above the interaction matrix. The gray scale of the squares indicates interaction strength between hosts and fungi, that is, black indicates the fungus was detected in all the five samples of the corresponding host, and the lightest gray association means the fungus was only found twice out of the five samples. The crosshatch cells show host–fungi associations for the three broad‐spectrum genera which were detected at least three times out of the five samples on more than 10 host species

Host–fungus associations for foliar fungi were highly clustered on the fungus phylogeny for all the hosts (Figures 2a and 3a). The relationships between hosts and foliar fungi were statistically nonrandom (ParaFit test: *p *=* *.0251 after 9,999 permutations), providing a clear support for phylogenetic congruence between hosts and their leaf‐associated fungi. The fungi that were detected in the same host were usually closely related, which contributed substantially to the nonrandom associations, and whenever a fungal genus infected two or more tree species, the host species were also found to be closely related (Figures 2a and 3a).

A congruent pattern of host phylogeny and fungus phylogeny was also detected for the soil‐borne fungi (i.e., closely related fungal species were associated with related tree species, Figures 2b and 3b). We obtained significant support for the hypothesis that there was global nonrandom association between subtropical trees and their soil fungi (ParaFit test: *p *=* *.0203). Almost all of the soil fungi were specific to one to five hosts that were closely related (Figures 2b and 3b), except for three broad‐spectrum genera *Aspergillus*,* Penicillium,* and *Cladophialophora*. These three genera were detected on more than 10 host species (13 ± 1.73) that were widely distributed on the host phylogenetic tree, which was significantly larger than for the overall network (3 ± 0.54, *p *=* *.02).

The two plant–fungus interaction networks had relative high modularity values (*M *=* *0.66 and 0.40 for foliar and soil fungal networks, respectively), but neither were statistically different from null models (*p *=* *.06 and .77, respectively), probably due to the relatively small size of the networks.

## 4 Discussion

By sequencing and identifying potential fungal pathogens associated with diseased leaves, rotten seeds, and infected seedlings of 26 tree species, we detected significantly nonrandom plant–fungal networks in a subtropical forest. The host–fungus combinations showed phylogenetic congruence on the fungal and host phylogenies, which supports the phylogenetic conservatism observed in plant–soil feedbacks (Brandt, Seabloom, & Hosseini, 2009; Liu et al., 2012) as well as the phenomenon that phylogenetic structure and host genetic variation shape disease pressure in plant communities (Busby, Newcombe, Dirzo, & Whitham, 2013; Gilbert & Webb, 2007; Liu et al., 2015; Parker et al., 2015). While these previous studies detecting significant phylogenetic signal in plant–fungal interactions treated the fungal community as a black box (Brandt et al., 2009; Liu et al., 2012), our study provides a direct, empirical view of the complex networks among subtropical trees and the fungal pathogens associated with their leaves, seeds, and roots. The fact that in a plant community, most pathogenic fungi within the same genus infect several closely related species from multiple genera and families suggests the Janzen–Connell effect caused by fungal microbes may be more effective at maintaining diversity at higher taxonomic levels than maintaining species diversity per se (Parker et al., 2015).

The concordant pattern in phylogenies could be caused by several different ecological processes. Because the sampled individuals were randomly selected across the 20‐ha area and not spatially autocorrelated or phylogenetically clustered, a plausible explanation is that host species coevolve with their plant‐associated fungi. Parallel patterns of phylogenetic trees have been found between host rodents and their lice (Hafner & Nadler, 1988) and between ants and fungi (Hinkle, Wetterer, Schultz, & Sogin, 1994), which suggests that these host–parasite assemblages have coevolved. Coevolution between hosts and their natural enemies, including viruses, fungi, bacteria, nematodes, insects, and mammals, is believed to have generated much of the Earth's biological diversity (Thompson, 2014). There is a growing appreciation among ecologists that long‐term evolutionary history has a major role in explaining the composition and structure of ecological assemblages or communities (see reviews in Emerson & Gillespie, 2008; Weber & Agrawal, 2012). Previous coevolution studies typically considered a single host species interacting with a single pathogen species (e.g., Laine, 2004; Thrall & Burdon, 2003). However, in reality, most host populations encounter a large number of different pathogen species, and most pathogen species can infect more than one species of host (Gilbert, Magarey, & Webb, 2012). Our study reveals phylogenetically structured, nonrandom networks among multiple fungi and multiple hosts, suggesting that the potential plant–fungus coevolution may proceed in a diffuse manner (Juenger & Bergelson, 1998); having multiple hosts may mean reduced selection pressure for a pathogen.

Another plausible explanation driving the congruent phylogenies is host tracking by the parasites. Plant traits commonly show a phylogenetic signal, including the ones that are important in plant–enemy interactions, where close relatives are more likely to have similar traits (Agrawal, 2007), and are thereby susceptible to colonization by closely related pathogens. Due to their limited dispersal ability, initial colonization of fungi is most likely to occur on spatially associated hosts (e.g., Packer & Clay, 2000). Closely related tree species may also favor similar environmental conditions such as light and soil moisture (Emerson & Gillespie, 2008), which could facilitate the growth and reproduction of some closely related fungi that also prefer the same environments (Horn, Caruso, Verbruggen, Rillig, & Hempel, 2014). Future research will be required to identify relative importance of the two explanations that caused the concordant pattern in phylogenies: While complementary data on microclimatic differences and host traits could test the host tracking process, accurate time‐calibrated phylogenies could be used to support the host–parasite coevolution explanation if the speciation events for both hosts and their associated fungi coincide.

Our ability to investigate complicated plant–fungus interaction networks in natural communities benefits from recent developments in molecular technology. Molecular identification through DNA barcoding of fungi has become an integral and essential part of fungal ecology research and has provided new insights into the diversity and ecology of many different groups of fungi (Anderson & Cairney, 2004; Bellemain et al., 2010). Molecular identification has made it possible to study the ecology of fungi in their dominant, but inconspicuous mycelium stage, and not only by observation of fruiting bodies or selective culturing techniques. Using high‐throughput sequencing, thousands of sequences can be analyzed from a single environmental sample, enabling researchers to undertake an in‐depth analysis of fungal diversity (Bellemain et al., 2010). However, we realize that although ITS combines the highest resolving power for discriminating closely related species and has a high sequencing success rate across a broad range of fungi (Schoch et al., 2012), it has some methodological limitations as functionally distinct fungi (e.g., pathogenic vs. mutualistic species) may have nearly identical ITS sequences. The species‐level identification of plant–fungus associations is also limited by incomplete databases of fungal diversity. Here, we identified effective host–fungus combinations when a fungal OTU was found in three or more samples among the five samples, which is a probabilistically reasonable but coarse method to distinguish potential fungal pathogens, given the complexity associated with many plant and fungal species in the field.

The two networks have relatively high modularity values, but neither were significant different from null models. This is presumably related to the small size of our host–fungus networks, as previous studies have usually found significant modularity in ecological networks with many species (283.9 ± 249.0, *N* = 29), but rarely in networks with few species (67.1 ± 37.0, *N* = 22) (Toju et al., 2014). Although we were not able to sample all the host species in the local community, we chose 26 tree species that are common in the study area, and that ranged from less to more common. Also, these focal species were widely distributed across the overall phylogeny of all tree species in the study area, and had a wide range of functional traits. Hence, we believe these species are a good representation of the overall community. We also conducted the ParaFit test while both hosts and fungi were grouped at the genus level, and the phylogenetically congruent patterns remained for both foliar and soil fungal networks (*p *=* *.0217 and .0361, respectively), because congeneric hosts were usually infected by the same or closely related fungi.

In summary, we detected significant nonrandom host–fungus combinations. The host–fungus associations clustered on both the fungus phylogeny and the plant phylogeny, suggesting that tree hosts may coevolve with their associated fungi in a diffuse manner or that fungal species tracked host species because of phylogenetic conservatism of host traits. The phylogenetic congruence promotes host‐specific effects of fungi on their plant hosts and supports the expected role of fungal microbes in promoting species coexistence and maintaining biodiversity of forest communities.

## Conflict of Interest

None declared.
