# Supplementary material for: Phylogenetic congruence between subtropical trees and their associated fungi
Source: Ecol Evol. 2016 Oct 26;6(23):8412–22. doi: 10.1002/ece3.2503 (PMC5167024; doi:10.1002/ece3.2503)
Supplement: Supplementary file 1 [file ECE3-6-8412-s001.docx]

**Supporting Information**

**Figure S1** Spatial map indicating relative locations of sampling individuals for all focal tree species within the 20-ha sampling area.

**Table S1** Literature review for the pathogenicity of leaf endophytic fungi isolated from subtropical tree leaves in Heishiding Nature Reserve.

**Table S2** Literature review for the pathogenicity of soil-borne fungi isolated around subtropical tree roots in Heishiding Nature Reserve.

Figure S1

| **Genus** | **Family** | **Order** | **Class** | **References*** | |  |
| --- | --- | --- | --- | --- | --- | --- |
| Alternaria | Pleosporaceae | Pleosporales | Dothideomycetes | Nishimura & Kohmoto 1983; Rotem 1994; Thrall *et al.* 2001 | |  |
| Capnodium | Capnodiaceae | Capnodiales | Dothideomycetes | Lindberg 1981; Reynolds 1999; Mwenje & Mguni 2001 | |  |
| Cladosporium | Mycosphaerellaceae | Capnodiales | Dothideomycetes | De Wit *et al.* 1986; Ackerveken *et al.* 1992 | |  |
| Coccomyces | Rhytismataceae | Rhytismatales | Leotiomycetes | Petersen & Cation 1950; Cation 1953 | |  |
| Colletotrichum | Phyllachoraceae | Phyllachorales | Sordariomycetes | Jeffries *et al.* 1990; Bailey *et al.* 1992; Freeman *et al.* 1998 | |  |
| Cyphellophora | Chaetothyriaceae | Chaetothyriales | Eurotiomycetes | Bittencourt *et al.* 2002 | |  |
| Devriesia | Teratosphaeriaceae | Capnodiales | Dothideomycetes | Crous *et al.* 2010 | |  |
| Dictyochaeta | Chaetosphaeriaceae | Chaetosphaeriales | Sordariomycetes | Parungao *et al.* 2002; Dalling 2005 | |  |
| Didymosphaeria | Didymosphaeriaceae | Pleosporales | Dothideomycetes | Yen *et al.* 1956; Dorworth 1990; Taylor *et al.* 2001 | |  |
| Emarcea | Xylariaceae | Xylariales | Sordariomycetes | Duong *et al.* 2004 | |  |
| Fusidium | Nectriaceae | Hypocreales | Sordariomycetes | Backus & Stowell 1953; Jong & Ch'en 1966 | |  |
| Glomerella | Glomerellaceae | Glomerellales | Sordariomycetes | Shear & Wood 1913; Rodriguez & Yoder 1987 | |  |
| Guignardia | Mycosphaerellaceae | Capnodiales | Dothideomycetes | Okane *et al.* 2001; Bonants *et al.* 2003; Baldassari *et al.* 2008 | |  |
| Helicoma | Tubeufiaceae | Pleosporales | Dothideomycetes | Ottmann *et al.* 2012 | |  |
| Hortaea | Teratosphaeriaceae | Capnodiales | Dothideomycetes | Chen *et al.* 2012 | |  |
| Lophiostoma | Lophiostomataceae | Pleosporales | Dothideomycetes | Hyde 1995; Halleen *et al.* 2005 | |  |
| Massarina | Lophiostomataceae | Pleosporales | Dothideomycetes | Hyde & Aptroot 1997; | |  |
| Microdiplodia | Botryosphaeriaceae | Botryosphaeriales | Dothideomycetes | Shreemali 1970; Shreemali 1973 | |  |
|  |  |  |  |  | |  |
| Microdochium | Hyponectriaceae | Xylariales | Sordariomycetes | Simpson *et al.* 2001; Glynn & Edwards 2010 | |  |
| Mycosphaerella | Mycosphaerellaceae | Capnodiales | Dothideomycetes | McDonald *et al.* 1991; Rivas *et al.* 2004; Stukenbrock *et al.* 2007 | |  |
| Paraconiothyrium | Montagnulaceae | Pleosporales | Dothideomycetes | Damm *et al.* 2008 | |  |
| Pestalotiopsis | Amphisphaeriaceae | Xylariales | Sordariomycetes | Venkatasubbaiah & Van Dyke 1991; Ding *et al.* 2008 | |  |
| Pezicula | Dermateaceae | Helotiales | Ascomycetes | Johansen 1949; Leibinger *et al.* 1997; Johnston *et al.* 2005 | |  |
| Phialea | Incertae_sedis | Incertae_sedis | Incertae_sedis | Kienholz & Cash 1937; Calvert & Muskett 2008 | |  |
| Phialophora | Herpotrichiellaceae | Chaetothyriales | Dothideomycetes | McKeen 1952; Ajello *et al.* 1974; Baker 1980 | |  |
| Phoma | Incertae_sedis | Pleosporales | Dothideomycetes | Reddy *et al.* 1998; Onfroy *et al.* 1999; Jasiński *et al.* 2009 | |  |
| Phomopsis | Valsaceae | Diaporthales | Sordariomycetes | Udayanga *et al.* 2011 | |  |
| Pilidiella | Schizoparmaceae | Diaporthales | Sordariomycetes | Michailides *et al.* 2010; Thomidis & Exadaktylou 2011 | |  |
| Pseudocercospora | Mycosphaerellaceae | Capnodiales | Dothideomycetes | Braun & Dick 2002; Kirk *et al.* 2008 | |  |
| Pseudorobillarda | Incertae_sedis | Incertae_sedis | Incertae_sedis | Uecker & Kulik 1986 | |  |
| Ramichloridium | Mycosphaerellaceae | Capnodiales | Dothideomycetes | Pont 1960; Arzanlou *et al.* 2007 | |  |
| Readeriella | Teratosphaeriaceae | Capnodiales | Dothideomycetes | Macauley & Thrower 1965; Damm *et al.* 2008 | |  |
| Rhizopycnis | Incertae_sedis | Incertae_sedis | Dothideomycetes | Armengol *et al.* 2003; Ghignone *et al.* 2003 | | |
| Rhytidhysteron | Patellariaceae | Patellariales | Dothideomycetes | Ohm *et al.* 2012 | | |
| Seiridium | Amphisphaeriaceae | Xylariales | Sordariomycetes | Chou 1990; Graniti *et al.* 1992 | | |
| Sporobolomyces | Incertae_sedis | Sporidiobolales | Microbotryomycetes | Bashi & Fokkema 1977; Koizumi 1986 | | |
| Teratosphaeria | Teratosphaeriaceae | Capnodiales | Dothideomycetes | Crous *et al.* 2008; Hunter *et al.* 2009; Chen *et al.* 2011 | | |
|  |  |  |  | |  |  |

**Table S1** Literature review for the pathogenicity of leaf endophytic fungi isolated from subtropical tree leaves in Heishiding Nature Reserve.

***References:**

1. Ackerveken GF, Kan JA, Wit PJ. 1992. Molecular analysis of the avirulence gene avr9 of the fungal tomato pathogen *Cladosporium fulvum* fully supports the gene-for-gene hypothesis. *Plant Journal* **2**: 359-366.
2. Ajello L, Georg LK, Steigbigel RT, Wang CJK. 1974. A case of phaeohyphomycosis caused by a new species of Phialophora. *Mycologia* **66**: 490-498.
3. Armengol J, Vicent A, Martínez‐Culebras P, Bruton BD, García‐Jiménez J. 2003. Identification, occurrence and pathogenicity of *Rhizopycnis vagum* on muskmelon in Spain. *Plant Pathology* **52**: 68-73.
4. Arzanlou M, Groenewald JZ, Gams W, Braun U, Shin HD, Crous PW. 2007. Phylogenetic and morphotaxonomic revision of Ramichloridium and allied genera. *Studies in Mycology* **58**: 57-93.
5. Backus MP, Stowell EA. 1953. A Fusidium disease of Xylaria in Wisconsin. *Mycologia* **45**: 836-847.
6. Bailey JA, O'Connell RJ, Pring RJ, Nash C, Jeger MJ. 1992. Infection strategies of *Colletotrichum* species. In: Bailey JA & Jeger MJ, eds. *Colletotrichum: Biology, pathology and control.* Wallingford, UK: CABI, 88-120.
7. Baker R. 1980. Measures to control Fusarium and Phialophora wilt pathogens of carnation. *Plant Disease* **64**: 743-749.
8. Baldassari RB, Wickert E, de Goes A. 2008. Pathogenicity, colony morphology and diversity of isolates of *Guignardia citricarpa* and *G. mangiferae* isolated from Citrus spp. *European Journal of Plant Pathology* **120**: 103-110.
9. Bashi E, Fokkema NJ. 1977. Environmental factors limiting growth of *Sporobolomyces roseus*, an antagonist of *Cochliobolus sativus*, on wheat leaves. *Transactions of the British Mycological Society* **68**: 17-25.
10. Bittencourt AL, Machado PR, Araujo MG. 2002. Subcutaneous phaeohyphomycosis caused by *Cyphellophora pluriseptata*. *European Journal of Dermatology* **12**: 103-106.
11. Bonants PJ, Carroll GC, de Weerdt M, van Brouwershaven IR, Baayen RP. 2003. Development and validation of a fast PCR-based detection method for pathogenic isolates of the citrus black spot fungus, *Guignardia citricarpa*. *European Journal of Plant Pathology* **109**: 503-513.
12. Braun U, Dick MA. 2002. Leaf spot diseases of eucalypts in New Zealand caused by Pseudocercospora species. *New Zealand Journal of Forestry Science* **32***:* 221-234.
13. Cation D. 1953. Experiments with Actidione for control of cherry leaf spot (*Coccomyces hiemalis*). *Phytopathology* **43**: 468.
14. Calvert EL, Muskett AE. 1945. Blind-seed disease of rye-grass (*Phialea temulenta* Prill. & Delacr.). *Annals of Applied Biology* **32**: 329-343.
15. Chen J, Xing XK, Zhang LC, Xing YM, Guo SX. 2012. Identification of *Hortaea werneckii* isolated from mangrove plant *Aegiceras comiculatum* based on morphology and rDNA sequences. *Mycopathologia* **174**: 457-466.
16. Chen SF, Barnes I, Chungu D, Roux J, Wingfield MJ, Xie YJ, Zhou XD. 2011. High population diversity and increasing importance of the Eucalyptus stem canker pathogen, Teratosphaeria zuluensis, in South China. *Australasian Plant Pathology* **40**: 407-415.
17. Chou CKS. 1990. Pathogenic variation of Seiridium spp. isolated from cankered Cuppressaceae hosts in New Zealand. *European Journal of Forest Pathology* **20**: 32-43.
18. Crous PW, Summerell BA, Mostert L, Groenewald JZ. 2008. Host specificity and speciation of Mycosphaerella and Teratosphaeria species associated with leaf spots of Proteaceae. *Persoonia: Molecular Phylogeny and Evolution of Fungi* **20**: 59.
19. Crous PW, Groenewald JZ, Shivas RG. 2010. *Devriesia fraseriae* Crous & RG Shivas, sp. nov. *Persoonia* **25**: 151.
20. Dalling JW. 2005. The fate of seed banks: factors influencing seed survival for light-demanding species in moist tropical forests. In: Forget PM, Lambert JE, Hulme PE, Vander Wall SB, eds. *Seed fate: Predation, dispersal, and seedling establishment.* Wallingford, UK: CABI, 31-44.
21. Damm U, Verkley GJM, Crous PW, Fourie PH, Haegi A, Riccioni L. 2008. Novel Paraconiothyrium species on stone fruit trees and other woody hosts. *Persoonia-Molecular Phylogeny and Evolution of Fungi* **20**: 9-17.
22. De Wit PJ, Buurlage MB, Hammond KE. 1986. The occurrence of host-, pathogen-and interaction-specific proteins in the apoplast of *Cladosporium fulvum* (syn. *Fulvia fulva*) infected tomato leaves. *Physiological and Molecular Plant Pathology* **29**: 159-172.
23. Ding G, Jiang L, Guo L, Chen X, Zhang H, Che Y. 2008. Pestalazines and pestalamides, bioactive metabolites from the plant pathogenic fungus *Pestalotiopsis theae*. *Journal of natural products* **71**: 1861-1865.
24. Dorworth CE. 1990. Employment of pathogens to constrain growth of undesirable forest vegetation. *Proceedings of the VIII International Symposium on Biological Control of Weeds*: 471-476.
25. Duong LM, Lumyong S, Hyde KD, Jeewon R. 2004. *Emarcea castanopsidicola* gen. et sp. nov. from Thailand, a new xylariaceous taxon based on morphology and DNA sequences. *Studies in Mycology* **50**: 253-260.
26. Freeman S, Katan T, Shabi E. 1998. Characterization of *Colletotrichum* species responsible for anthracnose diseases of various fruits. *Plant Disease* **82**: 596-605.
27. Ghignone S, Tamietti G, Girlanda M. 2003. Development of specific PCR primers for identification and detection of Rhizopycnis vagum. *European journal of Plant Pathology* **109**: 861-870.
28. Glynn NC, Edwards SG. 2010. Evaluation of PCR assays for quantifying seed‐borne infection by Fusarium and Microdochium seedling blight pathogens. *Journal of Applied Microbiology* **108**: 81-87.
29. Graniti A, Sparapano L, Evidente A. 1992. Cyclopaldic acid, a major phytotoxic metabolite of Seiridium cupressi, the pathogen of a canker disease of cypress. *Plant pathology* **41**: 563-568.
30. Halleen F, van Niekerk J, Mostert L, Fourie P, Crous P. 2005. Trunk disease pathogens associated with apparently healthy nursery grapevines. *Wineland (Wynboer) Technical Yearbook. Stellenbosch, South Africa*: 12-14.
31. Hunter GC, Crous PW, Carnegie AJ, Wingfield MJ. 2009. *Teratosphaeria nubilosa*, a serious leaf disease pathogen of Eucalyptus spp. in native and introduced areas. *Molecular Plant Pathology* **10:** 1-14.
32. Hyde KD. 1995. *Lophiostoma asiana* sp. nov. from Thailand mangroves. *Mycotaxon* **55**: 283-288.
33. Jasiński M, Kachlicki P, Rodziewicz P, Figlerowicz M, Stobiecki M. 2009. Changes in the profile of flavonoid accumulation in *Medicago truncatula* leaves during infection with fungal pathogen *Phoma medicaginis*. *Plant Physiology and Biochemistry* **47**: 847-853.
34. Jeffries P, Dodd JC, Jeger MJ, Plumbley RA. 1990. The biology and control of *Colletotrichum* species on tropical fruit crops. *Plant Pathology* **39**: 343-366.
35. Johansen G. 1949. The Danish species of the Discomycete Genus Pezicula. *Dansk Botanisk Arkiv* **13**: 26.
36. Johnston PR, Pennycook SR, Manning MA. 2005. Taxonomy of fruit-rotting fungal pathogens: what's really out there? *New Zealand Plant Protection* **58**: 42.
37. Jong SC, Ch'en CC. 1966. Survey of damping-off pathogens from coniferous seedlings in Taiwan. *Plant Protection Bulletin, Taiwan* **8**: 129-139.
38. Kienholz JR, Cash EK. 1937. A New Species of Phialea on Alder Seeds. *Mycologia* **29**: 81-84.
39. Kirk PM, Cannon PF, Minter DW, Stalpers JA. 2008. *Dictionary of the Fungi* (10th ed.). Wallingford, UK: CABI, 567.
40. Koizumi M. 1986. *Sporobolomyces roseus*, a causal agent of citrus pseudo greasy spot (Nise-ohan-byo) and the infection process of disease. *Annals of the Phytopathological Society of Japan* **52**: 758-765.
41. Leibinger W, Breuker B, Hahn M, Mendgen K. 1997. Control of postharvest pathogens and colonization of the apple surface by antagonistic microorganisms in the field. *Phytopathology* **87**: 1103-1110.
42. Lindberg GD. 1981. An Antibiotic Lethal to Fungi. *Plant Disease* **65**: 680-683.
43. Macauley BJ, Thrower LB. 1965. *Readeriella*, a little-known genus of Sphaeropsidales. *Transactions of the British Mycological Society* **48**: 105-111.
44. McDonald BA, Martinez JP. 1991. DNA fingerprinting of the plant pathogenic fungus *Mycosphaerella graminicola* (anamorph *Septoria tritici*). *Experimental Mycology* **15**: 146-158.
45. McKeen WE. 1952. Phialophora radicicola Cain, a corn rootrot pathogen. *Canadian Journal of Botany* **30**: 344-347.
46. Michailides TJ, Puckett R, Morgan D. 2010. Pomegranate decay caused by *Pilidiella granati* in California. *Phytopathology* **100**: S83.
47. Mwenje E, Mguni N. 2001. Cellulolytic and pectinolytic activities of *Capnodium* isolates (sooty mould) from Zimbabwe. *Canadian Journal of Botany* **79**: 1492-1495.
48. Nishimura S, Kohmoto K. 1983. Host-specific toxins and chemical structures from *Alternaria* species. *Annual Review of Phytopathology* **21**: 87-116.
49. Ohm RA, Feau N, Henrissat B, Schoch CL, Horwitz BA, Barry KW, Condon BJ, Copeland, AC, Dhillon B, Glaser F, et al. 2012. Diverse lifestyles and strategies of plant pathogenesis encoded in the genomes of eighteen *Dothideomycetes* fungi. *PLoS Pathogens* **8**: e1003037.
50. Okane I, Nakagiri A, Ito T. 2001. Identity of *Guignardia sp.* inhabiting ericaceous plants. *Canadian Journal of Botany* **79**: 101-109.
51. Onfroy C, Tivoli B, Corbiere R, Bouznad Z. 1999. Cultural, molecular and pathogenic variability of *Mycosphaerella pinodes* and *Phoma medicaginis* var. pinodella isolates from dried pea (*Pisum sativum*) in France. *Plant Pathology* **48***:* 218-229.
52. Ottmann C, van der Hoorn RA, Kaiser M. 2012. The impact of plant–pathogen studies on medicinal drug discovery. *Chemical Society Reviews* **41**: 3168-3178.
53. Parungao MM, Fryar SC, Hyde KD. 2002. Diversity of fungi on rainforest litter in North Queensland, Australia. *Biodiversity Conservation* **11**: 1185-1194.
54. Petersen D, Cation D. 1950. Exploratory experiments on the use of actidione for the control of Peach brown rot and Cherry leaf spot. *Plant Disease Reporter* **34**: 5-6.
55. Pont W. 1960. Three leaf speckle diseases of the Banana in Queensland. *Queensland Journal of Agricultural Science* **17**: 273-309.
56. Reddy PV, Patel R, White JrJF. 1998. Phylogenetic and developmental evidence supporting reclassification of cruciferous pathogens *Phoma lingam* and *Phoma wasabiae* in Plenodomus. *Canadian Journal of Botany* **76**: 1916-1922.
57. Reynolds DR. 1999. *Capnodium citri*: the sooty mold fungi comprising the taxon concept. *Mycopathologia* **148**: 141-147.
58. Rivas GG, Zapater MF, Abadie C, Carlier J. 2004. Founder effects and stochastic dispersal at the continental scale of the fungal pathogen of bananas *Mycosphaerella fijiensis*. *Molecular Ecology* **13**: 471-482.
59. Rodriguez RJ, Yoder OC. 1987. Selectable genes for transformation of the fungal plant pathogen *Glomerella cingulata* f. sp. *Phaseoli* (*Colletotrichum lindemuthianum*). *Gene* **54**: 73-81.
60. Rotem J. 1994. The Genus *Alternaria*: Biology, Epidemiology, and Pathogenicity. St Paul, Minnesota, USA: APS Press.
61. Shear CL, Wood AK. 1913. Studies of fungus parasites belonging to the genus *Glomerella*. *USDA Bureau of Plant Industry Bulletin* **252**: 110.
62. Shreemali JL. 1970. Three new leaf spot diseases caused by phytopathogenic fungi. *Journal of Applied Sciences* **2**: 58-60.
63. Shreemali JL. 1973. New pathogenic species of Microdiplodia from Muehlenbeckia platycladoes. *Indian journal of mycology and plant pathology* 120-121.
64. Simpson DR, Weston GE, Turner JA, Jennings P, Nicholson P. 2001. Differential control of head blight pathogens of wheat by fungicides and consequences for mycotoxin contamination of grain. *European Journal of Plant Pathology* **107**: 421-431.
65. Stukenbrock EH, Banke S, Javan-Nikkhah M, McDonald BA. 2007. Origin and domestication of the fungal wheat pathogen *Mycosphaerella graminicola* via sympatric speciation. *Molecular Biology and Evolution* **24**: 398-411.
66. Taylor JE, Crous PW, Swart L. 2001. Foliicolous and caulicolous fungi associated with Proteaceae cultivated in California. *Mycotaxon* **78**: 75-103.
67. Thomidis T, Exadaktylou E. 2011. First Report of Pilidiella granati on Pomegranate with Symptoms of Crown Rot in the Prefecture of Xanthi, Greece. *Plant Disease* **95**: 79-79.
68. Thrall PH, Burdon JJ, Bock CH. 2001. Short-term epidemic dynamics in the Cakile maritime - *Alternaria brassicicola* host-pathogen association. *Journal of Ecology* **89**: 723-735.
69. Udayanga D, Liu X, McKenzie EH, Chukeatirote E, Bahkali AH, Hyde KD. 2011. The genus Phomopsis: biology, applications, species concepts and names of common phytopathogens. *Fungal Diversity* **50**: 189-225.
70. Uecker FA, Kulik MM. 1986. Pseudorobillarda sojae, a new pycnidial coelomycete from soybean stems. *Mycologia* 78: 449-453.
71. Venkatasubbaiah P, Van Dyke CG. 1991. Phytotoxins produced by *Pestalotiopsis oenotherae*, a pathogen of evening primrose. *Phytochemistry* **30**: 1471-1474.
72. Yen WY, Chen MJ, Huang KT. 1956. Leaf scorch of Peanut (a new disease). *Journal of Agriculture and Forestry* **10**: 145-168.

**Table S2** Literature review for the pathogenicity of soil-borne fungi isolated around subtropical tree roots in Heishiding Nature Reserve.

| **Genus** | **Family** | | | **Order** | | **Class** | **References*** | |
| --- | --- | --- | --- | --- | --- | --- | --- | --- |
| Botryosphaeria | Botryosphaeriaceae | | | Botryosphaeriales | | Dothideomycetes | Arauz & Sutton 1989; Brown-Rytlewski & McManus 2000 | |
| Diplodia | Botryosphaeriaceae | | | Botryosphaeriales | | Dothideomycetes | Chou 1976; Stanosz *et al.* 2007 | |
| Lasiodiplodia | Botryosphaeriaceae | | | Botryosphaeriales | | Dothideomycetes | Burgess *et al.* 2006; Kindo *et al.* 2010; | |
| Neofusicoccum | Botryosphaeriaceae | | | Botryosphaeriales | | Dothideomycetes | Lazzizera *et al.* 2008; Espinoza *et al.* 2009 | |
| Phaeobotryon | Botryosphaeriaceae | | | Botryosphaeriales | | Dothideomycetes | Abdollahzadeh *et al.* 2009 | |
| Phyllosticta | Botryosphaeriaceae | | | Botryosphaeriales | | Dothideomycetes | Glienke C *et al.* 2011; Wikee *et al.* 2011 | |
| Capnodium | Capnodiaceae | | | Capnodiales | | Dothideomycetes | Reynolds 1999; Mwenje & Mguni 2001 | |
| Cercospora | Mycosphaerellaceae | | | Capnodiales | | Dothideomycetes | Lucas 1958; Neuhaus *et al.* 1991 | |
| Cladosporium | Mycosphaerellaceae | | | Capnodiales | | Dothideomycetes | Welty *et al.* 1968; Shimanuki 1987 | |
| Guignardia | Mycosphaerellaceae | | | Capnodiales | | Dothideomycetes | Kiely 1948; Bonants *et al*. 2003 | |
| Mycosphaerella | Mycosphaerellaceae | | | Capnodiales | | Dothideomycetes | Crous 2009; Stukenbrock *et al.* 2010 | |
| Batcheloromyces | Teratosphaeriaceae | | | Capnodiales | | Dothideomycetes | Taylor *et al.* 1999 | |
| Readeriella | Teratosphaeriaceae | | | Capnodiales | | Dothideomycetes | Barbed *et al.* 2003; Crous *et al.* 1989 | |
| Teratosphaeria | Teratosphaeriaceae | | | Capnodiales | | Dothideomycetes | Crous *et al.* 2008; Swart *et al.* 1998 | |
| Rhytidhysteron | Patellariaceae | | | Patellariales | | Dothideomycetes | Chowdhary *et al.* 2008; Ohm 2011 | |
| Corynespora | Corynesporascaceae | | | Pleosporales | | Dothideomycetes | Wei 1950; Chase 1982 | |
| Phoma | Incertae_sedis | | | Pleosporales | | Dothideomycetes | Hassan *et al.* 1991; Fitt *et al.* 2006 | |
| Pyrenochaeta | Incertae_sedis | | | Pleosporales | | Dothideomycetes | Goodenough & Mav 1973; Doganlar *et al.* 1998 | |
| Coniothyrium | Leptosphaeriaceae | | | Pleosporales | | Dothideomycetes | Ahmed & Tribe 1977; Gezahgne *et al.* 2005 | |
| Leptosphaeria | Leptosphaeriaceae | | | Pleosporales | | Dothideomycetes | Goodwin & Annis 1991; West *et al.* 2001 | |
| Alternaria | Pleosporaceae | | | Pleosporales | | Dothideomycetes | Nishimura *et al.* 1978; Johnson *et al.* 2000 | |
| Cochliobolus | Pleosporaceae | | | Pleosporales | | Dothideomycetes | Tzeng *et al.* 1992; Amadioha 2002 | |
| Leptosphaerulina | Pleosporaceae | | | Pleosporales | | Dothideomycetes | Thal & Campbell 1986; Barbetti 1995 | |
| Stemphylium | Pleosporaceae | | | Pleosporales | | Dothideomycetes | Heath & Higgins 1973; Borges *et al.* 1976 | |
|  |  | | |  | |  |  | |
| Fusicladium | Venturiaceae | | | Pleosporales | | Dothideomycetes | Linford 1926; Sánchez-Torres *et al.* 2009 | |
| Phaeocryptopus | Venturiaceae | | | Pleosporales | | Dothideomycetes | Manter *et al.* 2003; Winton *et al.* 2007 | |
| Phaeomoniella | Herpotrichiellaceae | | | Chaetothyriales | | Eurotiomycetes | Kakalíková *et al.* 2006; McLean *et al.* 2009 | |
| Aspergillus | Trichocomaceae | | | Eurotiales | | Eurotiomycetes | Geiser *et al.* 2000; Nierman *et al.* 2005 | |
| Byssochlamys | Trichocomaceae | | | Eurotiales | | Eurotiomycetes | Houbraken *et al.* 2006; Houbraken *et al.* 2008 | |
| Penicillium | Trichocomaceae | | | Eurotiales | | Eurotiomycetes | Hsieh *et al.* 1987; Baigre 2003 | |
| Verrucaria | Verrucariaceae | | | Verrucariales | | Eurotiomycetes | Belisario *et al.* 1999; Boyette *et al.* 2001 | |
| Chalara | Incertae_sedis | | | Incertae_sedis | | Incertae_sedis | Kowalski 2006; Halmschlager & Kirisits 2008 | |
| Geastrumia | Incertae_sedis | | | Incertae_sedis | | Ascomycetes | Johnson & Sutton 1994; Johnson *et al.* 1997 | |
| Pyriculariopsis | Incertae_sedis | | | Hypocreales | | Sordariomycetes | Malca & Owen 1957; Soares *et al.* 2011 | |
| Veronaea | Incertae_sedis | | | Incertae_sedis | | Ascomycetes | Tushemereirwe & Waller 1993; French 2006 | |
| Xylogone | Incertae_sedis | | | Incertae_sedis | | Incertae_sedis | Ja 1998; Kang *et al.* 2010 | |
| Medeolaria | Medeolariaceae | | | Medeolariales | | Incertae_sedis | LoBuglio *et al.* 2010;Lobuglio & Pfister 2013 | |
| Erysiphe | Erysiphaceae | | | Erysiphales | | Leotiomycetes | Ellingboe 1972; Ramonell *et al.* 2005 | |
| Cryptosporiopsis | Dermateaceae | | | Helotiales | | Leotiomycetes | Dugan *et al.* 1993; Sankaran *et al.* 1995 | |
| **Table S2** *Continued* | | | | | | | | |
| **Genus** | | **Family** | | **Order** | | **Class** | | **References** |
| Gloeosporium | | Dermateaceae | | Helotiales | | Leotiomycetes | | Edney 1956; Cho *et al.* 2003 |
| Mollisia | | Dermateaceae | | Helotiales | | Leotiomycetes | | Iurku 1978; Jaczewska-Kalicka 2002 |
| Hymenoscyphus | | Helotiaceae | | Helotiales | | Leotiomycetes | | Timmermann *et al.* 2011; Gross *et al.* 2012 |
| Idriella | | Helotiaceae | | Helotiales | | Leotiomycetes | | Nelson & Wilhelm 1956; Hodges & Campbell 1996 |
| Pezizella | | Helotiaceae | | Helotiales | | Leotiomycetes | | Weiss 1940; Crous *et al.* 1989 |
| Cadophora | | Incertae_sedis | | Helotiales | | Leotiomycetes | | Johnston *et al.* 2005; Chen *et al.* 2010 |
| Dactylaria | | Incertae_sedis | | Helotiales | | Leotiomycetes | | Kadir & Raghavan 1999; Kadir *et al.* 2009 |
| Gloeotinia | | Incertae_sedis | | Helotiales | | Leotiomycetes | | Hardison 1962; Alderman 1991 |
| Phacidiella | | Incertae_sedis | | Helotiales | | Leotiomycetes | | Hahn 1957; Funk 1969 |
|  | |  | |  | |  | |  |
| Rhexocercosporidium | | Incertae_sedis | | Helotiales | | Leotiomycetes | | Reeleder *et al.* 2006; Reeleder 2007 |
| Scytalidium | | Incertae_sedis | | Helotiales | | Leotiomycetes | | Barua *et al.* 2007; Kang *et al.* 2010 |
| Lanzia | | Rutstroemiaceae | | Helotiales | | Leotiomycetes | | Burpee & Goulty 1986; Carrow *et al.* 1987 |
| Scleromitrula | | Rutstroemiaceae | | Helotiales | | Leotiomycetes | | Kishi 1998; Hong *et al.* 2007 |
| Coccomyces | | Rhytismataceae | | Rhytismatales | | Leotiomycetes | | Petersen & Cation 1950; Cation 1953 |
| Lophodermium | | Rhytismataceae | | Rhytismatales | | Leotiomycetes | | Diwani & Millar 1987; Phillips & Burdekin 1992 |
| Galactomyces | | Dipodascaceae | | Saccharomycetales | | Saccharomycetes | | McKay 2011; Talibi *et al.* 2012 |
| Diaporthe | | Diaporthaceae | | Diaporthales | | Sordariomycetes | | Arimoto *et al.* 1986; Santos *et al*. 2011 |
| Cytospora | | Valsaceae | | Diaporthales | | Sordariomycetes | | Rozsnyay 1977; Royse & Ries 1978 |
| Ophiognomonia | | Valsaceae | | Diaporthales | | Sordariomycetes | | Broders 2011; Broders *et al.* 2012 |
| Phomopsis | | Valsaceae | | Diaporthales | | Sordariomycetes | | Mostert *et al.* 2000; Nita *et al.* 2006 |
| Valsa | | Valsaceae | | Diaporthales | | Sordariomycetes | | Abe *et al.* 2007; Wang *et al.* 2011 |
| Bionectria | | Bionectriaceae | | Hypocreales | | Sordariomycetes | | Bienapfl *et al.* 1700; Holguín-Peña *et al.* 2012 |
| Heteroepichloe | | Clavicipitaceae | | Hypocreales | | Sordariomycetes | | Tanaka *et al.* 2009 |
| Gliocephalotrichum | | Hypocreaceae | | Hypocreales | | Sordariomycetes | | Nishijima *et al.* 2002; Sakinah & Latiffah 2013 |
| Hypomyces | | Hypocreaceae | | Hypocreales | | Sordariomycetes | | Moreau *et al.* 1948; Filer 1967 |
| Geosmithia | | Incertae_sedis | | Hypocreales | | Sordariomycetes | | Freeland *et al.* 2009; Tisserat *et al.* 2011 |
| Myrothecium | | Incertae_sedis | | Hypocreales | | Sordariomycetes | | Bean *et al.* 1984; Boyette *et al.* 2001 |
| Calonectria | | Nectriaceae | | Hypocreales | | Sordariomycetes | | Booth 1971; Lombard *et al.* 2010 |
| Cylindrocarpon | | Nectriaceae | | Hypocreales | | Sordariomycetes | | Unestam *et al.* 1989; Rahman & Punja 2005 |
| Cylindrocladiella | | Nectriaceae | | Hypocreales | | Sordariomycetes | | Scattolin & Montecchio 2007; Inderbitzin *et al.* 2012 |
| Fusarium | | Nectriaceae | | Hypocreales | | Sordariomycetes | | Snyder & Hansen 1940; Sutton 1982 |
| Fusidium | | Nectriaceae | | Hypocreales | | Sordariomycetes | | Backus & Stowell 1953; Jong & Ch'en 1966 |
| Gibberella | | Nectriaceae | | Hypocreales | | Sordariomycetes | | Schroeder & Christensen 1963; Bai & Shaner 2004 |
| Nectria | | Nectriaceae | | Hypocreales | | Sordariomycetes | | Ehrlich 1934; Manion *et al.* 1967 |
| Apiospora | | Apiosporaceae | | Incertae_sedis | | Sordariomycetes | | Zhang 2000; Crous & Groenewald 2013 |
| Glomerella | | Glomerellaceae | | Incertae_sedis | | Sordariomycetes | | Ogbebor *et al.* 2007; Rodriguez & Yoder 1987 |
| Gaeumannomyces | | Magnaporthaceae | | Incertae_sedis | | Sordariomycetes | | Graham 1951; Thomashow & Weller 1988 |
| Magnaporthe | | Magnaporthaceae | | Incertae_sedis | | Sordariomycetes | | Mitchell & Dean 1995; Dean *et al.* 2005 |
| Mycoleptodiscus | | Magnaporthaceae | | Incertae_sedis | | Sordariomycetes | | Shearer 1996; Watanabe *et al.* 1997 |
| Verticillium | | Plectosphaerellaceae | | Incertae_sedis | | Sordariomycetes | | Nazar *et al.* 1991; Barbara & Clewes 2003 |
| Sphaeronaemella | | Incertae_sedis | | Microascales | | Sordariomycetes | | Vakili 1985; Wu & Zheng 2011 |
| Grosmannia | | Ophiostomataceae | | Ophiostomatales | | Sordariomycetes | | Alamouti *et al.* 2011; Linnakoski *et al.* 2012 |
| **Table S2** *Continued* | | | | | | | | |
| **Genus** | | | **Family** | | **Order** | **Class** | | **References** |
| Ophiostoma | | | Ophiostomataceae | | Ophiostomatales | Sordariomycetes | | Gibbs *et al.* 1977; Brasier 1991 |
| Colletotrichum | | | Phyllachoraceae | | Phyllachorales | Sordariomycetes | | Smith & Black 1990; Hyde *et al.* 2009 |
| Pestalotiopsis | | | Amphisphaeriaceae | | Xylariales | Sordariomycetes | | Jeewon *et al.* 2004; Zhang *et al.* 2010 |
| Eutypa | | | Diatrypaceae | | Xylariales | Sordariomycetes | | Moller & Kasimatis 1978; Carter 1991 |
| Biscogniauxia | | | Xylariaceae | | Xylariales | Sordariomycetes | | Granata & Sidoti 2004; Jurc & Ogris 2006 |
| Hypoxylon | | | Xylariaceae | | Xylariales | Sordariomycetes | | Copony & Barnes 1974; Anderson *et al.* 1956 |
| Xylaria | | | Xylariaceae | | Xylariales | Sordariomycetes | | Rogers 1984; Horst 2001 |
| Coniophora | | | Coniophoraceae | | Boletales | Agaricomycetes | | Schmidhalter *et al.* 1993; Lee *et al.* 2004 |
| Ceratobasidium | | | Ceratobasidiaceae | | Cantharellales | Agaricomycetes | | Murray & Burpee 1984; Hietala *et al.* 2001 |
| Laetisaria | | | Corticiaceae | | Corticiales | Agaricomycetes | | Stalpers & Loerakker 1982; Hims *et al.* 1984 |
| Resinicium | | | Meruliaceae | | Polyporales | Agaricomycetes | | Connolly & Jellison 1995; Rizzo & Harrington 1988 |
| Phanerochaete | | | Phanerochaetaceae | | Polyporales | Agaricomycetes | | Bumpus *et al.* 1985; Mileski *et al.* 1988 |
| Pseudolagarobasidium | | | Phanerochaetaceae | | Polyporales | Agaricomycetes | | Hallenberg *et al.* 2008; Nakasone & Lindner 2012 |
| Irpex | | | Steccherinaceae | | Polyporales | Agaricomycetes | | Gilbertson & Ryvarden 1986; Novotný *et al.* 2000 |
| Junghuhnia | | | Steccherinaceae | | Polyporales | Agaricomycetes | | Hatakka 1994; Kile & Keane 2000 |
|  | | |  | |  |  | |  |
| Laxitextum | | | Stereaceae | | Russulales | Agaricomycetes | | Stavishenko *et al.* 2002; Prasher & Ashok 2013 |
| Stereum | | | Stereaceae | | Russulales | Agaricomycetes | | Shimizu *et al.* 2001; Shimizu *et al.* 2005 |
| Puccinia | | | Pucciniaceae | | Pucciniales | Pucciniomycetes | | Hawker 1966; Roy *et al.* 1998 |
| Rhizophydium | | | Rhizophydiaceae | | Rhizophydiales | Chytridiomycetes | | McKenzie & Morrall 1973; Hickman & Ho 2003 |
| Choanephora | | | Choanephoraceae | | Mucorales | Incertae_sedis | | Barnett & Lilly 1950; Siddiqui*et al.* 2009 |
| Cunninghamella | | | Cunninghamellaceae | | Mucorales | Incertae_sedis | | Cherian 2005; Saroj *et al.* 2012 |
| Rhizopus | | | Mucoraceae | | Mucorales | Incertae_sedis | | Wilson *et al.* 1987; Qing & Tian 2000 |

***References:**

1. Abe K, Kotoda N, Kato H, Soejima J. 2007. Resistance sources to Valsa canker (Valsa ceratosperma) in a germplasm collection of diverse Malus species. *Plant Breeding* **126**: 449-453.
2. Ahmed AHM, Tribe HT. 1977. Biological Control of White Rot of Onion (*Sclerotium cepivarum*) by *Coniothyrium minitans*. *Plant pathology* **26**: 75-78.
3. Alamouti SM, Wang V, DiGuistini S, Six DL, Bohlmann J, Hamelin RC, Feau N, Breuil C. 2011. Gene genealogies reveal cryptic species and host preferences for the pine fungal pathogen Grosmannia clavigera. *Molecular Ecology***20**: 2581-2602.
4. Alderman SC. 1991. Assessment of ergot and blind seed diseases of grasses in the Willamette Valley of Oregon. *Plant disease* **75**: 1038-1041.
5. Amadioha AC. 2002. Fungitoxic effects of extracts of Azadirachta Indica against Cochliobolus miyabeanus causing brown spot disease of rice. *Archives of Phytopathology and Plant protection* **35**: 37-42.
6. Anderson RL, Anderson GW, Schipper AL. 1956. *Hypoxylon canker of aspen*. US Department of Agriculture, Forest Service.
7. Arimoto Y, Homma Y, Misato T. 1986. Studies on citrus melanose and citrus stem-end rot by Diaporthe citri (Faw.) Wolf., 4: Antifungal substance in melanose spot. *Annals of the Phytopathological Society of Japan* 52: 39-46.
8. Backus MP, Stowell EA. 1953. A Fusidium disease of Xylaria in Wisconsin. *Mycologia* **45**: 836-847.
9. Bai G, Shaner G. 2004. Management and resistance in wheat and barley to Fusarium head blight. *Annual Review of Phytopathology* **42**: 135-161
10. Barbara DJ, Clewes E. 2003. Plant pathogenic Verticillium species: how many of them are there? *Molecular Plant Pathology* **4**: 297-305.
11. Barbetti MJ. 1995. Resistance in annual Medicago species to Phoma medicaginis and Leptosphaerulina trifolii under field conditions. *Animal Production Science* **35**: 209-214.
12. Barbed PA, Smith IW, Keane PJ. 2003. Foliar diseases of Eucalyptus spp. grown for ornamental cut foliage. *Australasian Plant Pathology* **32:** 109-111.
13. Barnett H, Lilly V. 1950. Influence of nutritional and environmental factors upon asexual reproduction of Choanephora cucurbitarum in culture. *Phytopathology* **40**: 80-89.
14. Barua P, Barua S, Borkakoty B, Mahanta J. 2007. Onychomycosis by Scytalidium dimidiatum in green tea leaf pluckers: report of two cases. *Mycopathologia* **164**: 193–5.
15. Baigre B. 2003. *Taints and Off Flavours in Foods*. CRC Press.
16. Bean GA, Fernando T, Jarvis BB, Bruton B. 1984. The isolation and identification of trichothecene metabolites from a plant pathogenic strain of *Myrothecium roridum*. *Journal of natural products* **47**: 727-729.
17. Belisario A, Forti E, Corazza L, Van Kesteren HA. 1999. First report of Myrothecium verrucaria from muskmelon seeds. *Plant Disease* **83**: 589-589.
18. Bienapfl JC, Floyd CM, Percich JA, Malvick DK. 2012. First Report of *Clonostachys rosea* Causing Root Rot of Soybean in the United States. *Plant Disease* **96**: 1700.
19. Booth C. 1971. *The genus Fusarium*. Kew, England: Commonwealth Mycological Institute.
20. Bonants PJ, Carroll GC, de Weerdt M, van Brouwershaven IR, Baayen RP. 2003. Development and validation of a fast PCR-based detection method for pathogenic isolates of the citrus black spot fungus, *Guignardia citricarpa*. *European Journal of Plant Pathology* **109**: 503-513.
21. Borges OL, Stanford EH, Webster RK. 1976. The host-pathogen interaction of alfalfa and Stemphylium botryosum. *Phytopathology* **66**: 749-753.
22. Boyette CD, Abbas HK, Walker HL. 2001. Control of kudzu with a fungal pathogen derived from Myrothecium verrucaria. *U.S. Patent* **6**: 274,
23. Broders K. 2011. Population structure of Ophiognomonia clavigignenti-juglandacearumreveals multiple introductions of the butternut canker fungus into North America. In: *APS-IPCC Joint Meeting*. Apsnet.
24. Broders KD, Boraks A, Sanchez AM, Boland GJ. 2012. Population structure of the butternut canker fungus, Ophiognomonia clavigignenti‐juglandacearum, in North American forests. *Ecology and Evolution* **2**: 2114-2127.
25. Bumpus JA, Tien M, Wright D, Aust SD. 1985. Oxidation of persistent environmental pollutants by a white rot fungus. *Science* **228**: 1434-1436.
26. Burpee LL, Goulty LG. 1986. Evaluation of two dollarspot forecasting systems for creeping bentgrass. *Canadian journal of plant science* **66**: 345-351.
27. Carrow RN, Johnson BJ, Burns RE. 1987. Thatch and quality of Tifway bermudagrass turf in relation to fertility and cultivation. *Agronomy journal***79**: 524-530.
28. Carter MV. 1991. *The status of Eutypa lata as a pathogen*. CAB International.
29. Cation D. 1953. Experiments with Actidione for control of cherry leaf spot (*Coccomyces hiemalis*). *Phytopathology* **43**: 468.
30. Chase AR. 1982. Corynespora leaf spot of Aeschynanthus pulcher and related plants. *Plant Disease* **66**:739-740.
31. Chen J, Dong HL, Meng ZX, Guo SX. 2010. Cadophora malorum and Cryptosporiopsis ericae isolated from medicinal plants of the Orchidaceae in China. *Mycotaxon* **112**: 457-461.
32. Cherian TT. 2005. Soft rot of cucumber (*Cucumis sativus* L.) by *Cunninghamella echinulata* Thaxt. *Journal of Mycopathological Research*, 43: 137-138.
33. Cho SJ, Lee SK, Cha BJ, Kim YH, Shin KS. 2003. Detection and characterization of the Gloeosporium gloeosporioides growth inhibitory compound iturin A from Bacillus subtilis strain KS03. *FEMS Microbiology Letters* **223**: 47-51.
34. Chowdhary A, Guarro J, Randhawa HS, Gené J, Cano J, Jain RK, Kumar S, Khanna G. 2008. A rare case of chromoblastomycosis in a renal transplant recipient caused by a non-sporulating species of Rhytidhysteron. *Medical Mycology* **46**: 163-166.
35. Connolly JH, Jellison J. 1995. Calcium translocation, calcium oxalate accumulation, and hyphal sheath morphology in the white-rot fungus Resinicium bicolor. *Canadian Journal of Botany* **73**: 927-936.
36. Copony JA, Barnes BV. 1974. Clonal variation in the incidence of Hypoxylon canker on trembling aspen. *Canadian Journal of Botany* **52**: 1475-1481.
37. Crous PW. 2009. Taxonomy and phylogeny of the genus Mycosphaerella and its anamorphs. *Fungal Diversity* **38**: 1-24.
38. Crous PW, Knox-Davies PS, Wingfield MJ. 1989. A list of Eucalyptus leaf fungi and their potential importance to South African forestry. *South African Forestry Journal* **149**: 17-29.
39. Crous PW, Summerell BA, Mostert L, Groenewald JZ. 2008. Host specificity and speciation of Mycosphaerella and Teratosphaeria species associated with leaf spots of Proteaceae. *Persoonia: Molecular Phylogeny and Evolution of Fungi* **20**: 59-86.
40. Crous PW, Groenewald JZ. 2013. A phylogenetic re-evaluation of Arthrinium. *IMA fungus* **4**: 133.
41. Dean RA, Talbot NJ, Ebbole DJ, Farman ML, Mitchell TK, Orbach MJ, Thon M, Kulkarni R, Xu JR, Pan H, *et al*. (2005). The genome sequence of the rice blast fungus Magnaporthe grisea. *Nature* **434**: 980-986.
42. Diwani SA, Millar CS. 1987. Pathogenicity of three Lophodermium species on Pinus sylvestris L. *European Journal of Forest Pathology* **17**: 53-58.
43. Doganlar S, Dodson J, Gabor B, Beck-Bunn T, Crossman C, Tanksley SD. 1998. Molecular mapping of the py-1 gene for resistance to corky root rot (*Pyrenochaeta lycopersici*) in tomato. *Theoretical and Applied Genetics* **97**: 784-788.
44. Dugan FM, Grove GG, Rogers JD. 1993. Comparative studies of Cryptosporiopsis curvispora and C. perennans. I. Morphology and pathogenic behavior. *Mycologia* **85**: 551-564.
45. Edney KL. 1956. The rotting of apples by Gloeosporium perennans Zeller & Childs. *Annals of Applied Biology* **44**: 113-128.
46. Ellingboe AH. 1972. Genetics and physiology of primary infection by Erysiphe graminis. *Phytopathology* **62**: 401.
47. Ehrlich J. 1934. The beech bark disease: a Nectria disease of Fagus, following Cryptococcus fagi (Baer.). *Canadian Journal of Research* **10**: 593-692.
48. Filer TH. 1967. Pathogenicity of Cytospora, Phomopsis, and Hypomyces on Populus deltoides. *Phytopathology* **57**: 978-980.
49. Fitt BDL, Brun H, Barbetti MJ, Rimmer SR. 2006. World-wide importance of phoma stem canker (*Leptosphaeria maculans* and *L. biglobosa*) on oilseed rape (*Brassica napus*). *European Journal of Plant Pathology* **114**: 3-15.
50. Holguín-Peña RJ, Hernández-Montiel LG, Latisnere H, Rueda-Puente EO. 2012. First Report of a Bionectria sp. Associated with a Stem Rot of Cardon Cactus (*Pachycereus pringlei*) in Baja California Sur, Mexico. *Plant Disease* **96**: 292-292.
51. Freeland E, Kolarik M, Utley C, Cranshaw W, Tisserat N. 2009. The Geosmithia causing thousand cankers disease of walnut is a new species. *Phytopathology* **99**: S37-S37.
52. Funk A. 1969. Potebniamyces (Phacidiella) disease of the true firs in British Columbia. *Canadian Journal of Botany* **47**: 751-753.
53. Geiser DM, Dorner JW, Horn BW, Taylor JW. 2000. The Phylogenetics of Mycotoxin and Sclerotium Production in *Aspergillus flavus* and *Aspergillus oryzae*. *Fungal Genetics and Biology* **31**: 169-179.
54. Gezahgne A, Cortinas MN, Wingfield MJ, Roux J. 2005. Characterisation of the Coniothyrium stem canker pathogen on Eucalyptus camaldulensis in Ethiopia. *Australasian Plant Pathology* **34**: 85-90.
55. Gibbs JN. 1978. Intercontinental epidemiology of Dutch elm disease. *Annual Review of Phytopathology* **16**: 287-307.
56. Gilbertson RL, Ryvarden L. 1986. North American polypores Vol. 1. Abortiporus to Lindtneria. Oslo, Fungiflora.
57. Goodenough PW, Maw GA. 1973. Effects of Pyrenochaeta lycopersici infection on nutrient uptake by tomato plants. *Annals of Applied Biology* **73**: 339-347.
58. Goodwin PH, Annis SL. 1991. Rapid identification of genetic variation and pathotype of Leptosphaeria maculans by random amplified polymorphic DNA assay. *Applied and Environmental Microbiology* **57**: 2482-2486.
59. Granata G., Sidoti A. 2004. Biscogniauxia nummularia: pathogenic agent of a beech decline. *Forest Pathology* 34: 363-367.
60. Gross A, Zaffarano PL, Duo A, Grünig CR. 2012. Reproductive mode and life cycle of the ash dieback pathogen *Hymenoscyphus pseudoalbidus. Fungal Genetics and Biology***49**: 977-986.
61. Hahn GG. 1957. A new species of Phacidiella causing the so-called Phomopsis disease of conifers. *Mycologia* **49**: 226-239.
62. Hallenberg N, Ryberg M, Nilsson RH, Wood AR, Wu SH. 2008. Pseudolagarobasidium (Basidiomycota): on the reinstatement of a genus of parasitic, saprophytic, and endophytic resupinate fungi. *Botany* **86**: 1319-1325.
63. Halmschlager E, Kirisits T. 2008. First report of the ash dieback pathogen Chalara fraxinea on Fraxinus excelsior in Austria. *Plant Pathology* **57**: 1177-1177.
64. Hassan AK, Schulz C, Sacristan MD, Wöstemeyer J. 1991. Biochemical and Molecular Tools for the Differentiation of Aggressive and Non‐Aggressive Isolates of the Oilseed Rape Pathogen, Phoma lingam. *Journal of Phytopathology* **131**: 120-136.
65. Hatakka A. 1994. Lignin-modifying enzymes from selected white-rot fungi: production and role from in lignin degradation. *FEMS Microbiology Reviews* **13**: 125-135.
66. Hawker, L. E. 1966. *Fungi: an* introduction. doi:10.1086/405552.
67. Heath MC, Higgins VJ. 1973. *In vitro* and *in vivo* conversion of phaseollin and pisatin by an alfalfa pathogen *Stemphylium botryosum*. *Physiological Plant Pathology* **3**: 107-120.
68. Hickman CJ, Ho HH. 2003. Behaviour of zoospores in plant-pathogenic Phycomycetes. *Annual Review of Phytopathology* **4**: 195-214.
69. Hietala AM, Vahala J, Hantula J. 2001. Molecular evidence suggests that *Ceratobasidium bicorne* has an anamorph known as a conifer pathogen. *Mycological Research* **105**: 555-562.
70. Hims MJ, Dickinson CH, Fletcher JT. 1984. Control of red thread, a disease of grasses caused by Laetisaria fuciformis. *Plant pathology* **33**: 513-516.
71. Hodges CF, Campbell DA. 1996. Infection of Adventitious Roots of Agrostis palustris by Idriella bolleyi1. *Journal of Phytopathology* **144**: 265-271.
72. Hong SK, Kim WG, Sung GB, Nam SH. 2007. Identification and distribution of two fungal species causing sclerotial disease on mulberry fruits in korea. *Mycobiology* **35**: 87-90.
73. Horst RK. 2001. Plant diseases and their pathogens. In: Horst RK, eds. *Westcott’s Plant Disease Handbook*. Springer US, 65-530
74. Houbraken J, Samson RA, Frisvad JC. 2006. Byssochlamys: significance of heat resistance and mycotoxin production. *Advances in Food Mycology* **571**: 211-224.
75. Houbraken J, Varga J, Rico-Munoz E, Johnson S, Samson RA. 2008. Sexual reproduction as the cause of heat resistance in the food spoilage fungus *Byssochlamys spectabilis* (anamorph *Paecilomyces variotii*). *Applied and Environmental Microbiology* **74**: 1613-1619.
76. Hsieh HM, Su HJ, Tzean SS. 1987. The genus Penicillium in Taiwan. I. Two new taxa of synnematous Penicillium. Transactions of the mycological society of republic of China. *Transactions of the mycological society of republic of China* **2**: 157-168.
77. Hyde KD, Cai L, Cannon PF, Crouch JA, Crous PW, Damm U, Goodwin PH, Chen H, Johnston PR, Jones, EBG, et al. 2009. Colletotrichum - names in current use. *Fungal Diversity* **39**: 147.
78. Inderbitzin P, Bostock RM, Subbarao KV. 2012. *Cylindrocladiella hahajimaensis*, a new species of Cylindrocladiella transferred from Verticillium. *MycoKeys* **4**: 1-8.
79. IUrku AI. 1978. Effect of fungicides on the spore formation process of Mollisia vitis, pathogen of grape spot necrosis. *Izvestiia Akademii nauk Moldavskoi SSR: seriia biologicheshikh i khimicheskikh nauk* **3**: 84-86.
80. Ja CG. 1998. Selection of effective fungicides against *Xylogone sphaerospora*, a fungal pathogen of cultivated mushroom, *Ganoderma lucidum*. *Korean Journal of Plant Pathology* **14**: 1.
81. Jaczewska-Kalicka A. 2002. The dominant pathogenic fungi on winter wheat in 1999-2001. *Acta Agrobotaica* **55**: 89-96.
82. Jeewon R, Liew ECY, Hyde KD. 2004. Phylogenetic evaluation of species nomenclature of Pestalotiopsis in relation to host association. *Fungal Diversity***17**: 39-55.
83. Johnson RD, Johnson L, Kohmoto K, Otani H, Lane CR, Kodama M. 2000. A polymerase chain reaction-based method to specifically detect Alternaria alternata apple pathotype (A. mali), the causal agent of Alternaria blotch of apple. *Phytopathology* **90**: 973-976.
84. Johnston PR, Pennycook SR, Manning MA. 2005. Taxonomy of fruit-rotting fungal pathogens: what's really out there? *New Zealand Plant Protection* **58**: 42.
85. Johnson EM, Sutton TB. 1994. First report of Geastrumia polystigmatis on apple and common blackberry in North America. *Plant Disease* **78**: 1219.
86. Johnson EM, Sutton TB, Hodges CS. 1997. Etiology of apple sooty blotch disease in North Carolina. *Phytopathology* **87**: 88-95.
87. Jong SC, Ch'en CC. 1966. Survey of damping-off pathogens from coniferous seedlings in Taiwan. *Plant Protection Bulletin, Taiwan* **8**: 129-39.
88. Jurc D, Ogris N. 2006. First reported outbreak of charcoal disease caused by Biscogniauxia mediterranea on Turkey oak in Slovenia. *Plant Pathology* **55**: 299-299.
89. Kadir JB, Charudattan R, Berger RD. 2009. Effects of some epidemiological factors on levels of disease caused by *Dactylaria higginsii* on *Cyperus rotundus*. *Weed Science* **48**: 61-68.
90. Kakalíková L, Jankura E, Šrobárová A. 2006. Phaeomoniella chlamydospora: causal agent of vine decline (*Vitis vinifera*) in the vineyards of Slovakia. *Plant Pathology* **55**: 815.
91. Kang HJ, Sigler L, Jungkwan L, Gibas CFC, Yun SH, Lee YW. 2010. Xylogone ganodermophthora sp. nov., an ascomycetous pathogen causing yellow rot on cultivated mushroom Ganoderma lucidum in Korea. *Mycologia* **102**: 1167-1184.
92. Kang HJ, Sigler L, Lee J, Gibas CFC, Yun SH, Lee YW. 2010. *Xylogone ganodermophthora* sp. nov., an ascomycetous pathogen causing yellow rot on cultivated mushroom *Ganoderma lucidum* in Korea. *Mycologia* 102: 1167-1184.
93. Kiely TB. 1948. Preliminary studies on *Guignardia citricarpa*, n. sp.: the ascigerous stage of *Phoma citricarpa* McAlp. and its relation to black spot of Citrus. *Proceedings of the Linnean Society of New South Wales* **73**: 249-292).
94. Kile GA, Keane PJ. 2000. Woody root rots of eucalypts. In: Kean PJ, Kile GA, Podger FD, Brown BN, eds*. Diseases and pathogens of eucalypts*, 293-306.
95. Kishi K. 1998. *Plant diseases in Japan*. Tokyo, Japan: Zenkoku-Noson-Kyoiku Kyokai Publishing.
96. Kowalski T. 2006. *Chalara fraxinea* sp. nov. associated with dieback of ash (*Fraxinus excelsior*) in Poland. *Forest Pathology***36**: 264-270.
97. Lee KH, Wi SG, Singh AP, Kim YS. 2004. Micromorphological characteristics of decayed wood and laccase produced by the brown-rot fungus Coniophora puteana. *Journal of Wood Science* **50**: 281-284.
98. Liqin Z. 2000. Recent situation and control of bamboo diseases in China. *Indian Journal of Forestry* **23**: 104-109.
99. Linford M. 1926. Black-leaf of Peas caused by *Fusicladium pisi-cola* n. sp. *Phytopathology* **16**: 549-558.
100. Linnakoski R, de Beer ZW, Duong TA, Niemelä P, Pappinen A, Wingfield MJ. 2012. Grosmannia and Leptographium spp. associated with conifer-infesting bark beetles in Finland and Russia, including Leptographium taigense sp. nov. *Antonie van Leeuwenhoek* **102**: 375-399.
101. LoBuglio KF, Donald HP. 2010. Placement of Medeolaria farlowii in the Leotiomycetes, and comments on sampling within the class. *Mycological Progress* **9**: 361-368.
102. Lobuglio KF, Pfister DH. 2013. Systemic Infection of *Medeola virginiana* (Liliaceae) by the Fungus *Medeolaria farlowii* (Ascomycota: Leotiomycetes). *Mycosystema* **32**: 342-346.
103. Lombard L, Crous PW, Wingfield BD, Wingfield MJ. 2010. Species concepts in Calonectria (Cylindrocladium). *Studies in Mycology* **66**: 1-13.
104. Lucas GB. 1958. *Diseases of tobacco.* Biological Consulting Associates, Raleigh.
105. Malca I, Owen JH. 1957. The gray leaf spot disease of St. Augustinegrass. *Plant Disease Reports* **41**: 871-875.
106. Manion PD, French DW. 1967. Nectria galligena and Ceratocystis fimbriata cankers of aspen in Minnesota. *Forest Science* **13**: 23-28.
107. Manter DK, Bond BJ, Kavanagh KL, Stone JK, Filip GM. 2003. Modelling the impacts of the foliar pathogen, *Phaeocryptopus gaeumannii*, on Douglas-fir physiology: net canopy carbon assimilation, needle abscission and growth. *Ecological Modelling* **164**: 211-226.
108. McKay AH. 2011. Population Structure of the Sour Rot Pathogens *Galactomyces citri-aurantii* and *G. geotrichum* and Evaluation of Sterol Demethylation Inhibitors for Postharvest Management of Citrus Decays. University of California.
109. McKenzie DL, Morrall RAA. 1973. Diseases of three specialty legume crops in Saskatchewan in 1972: field pea, lentil and fababean. *Canadian Plant Disease Survival* **53**: 187-190.
110. McLean T, Fourie PH, McLeod A. 2009. Reporter gene transformation of the trunk disease pathogen *Phaeomoniella chlamydospora* and biological control agent *Trichoderma harzianum*. *Australasian Plant Pathology* **38**: 153-167.
111. Mileski GJ, Bumpus JA, Jurek MA, Aust SD. 1988. Biodegradation of pentachlorophenol by the white rot fungus *Phanerochaete chrysosporium*. *Applied and Environmental Microbiology* **54**: 2885-2889.
112. Mitchell TK, Dean RA. 1995. The cAMP-dependent protein kinase catalytic subunit is required for appressorium formation and pathogenesis by the rice blast pathogen Magnaporthe grisea. *Plant Cell* **7**: 1869-1878.
113. Sakinah MAI, Latiffah Z. 2013. First report of *Gliocephalotrichum bacillisporum* causing fruit rot of rambutan (*Nephelium lappaceum* L.) in Malaysia. *Plant Disease* **97**: 1110.
114. Moller WJ, Kasimatis AN. 1978. Dieback of grapevines caused by *Eutypa armeniacae*. *Plant Disease Reporter* **62**: 254-258.
115. Moreau C, Moreau M, Jacquiot C. 1948. A canker disease of *Aucoumea klaineana* in Gabon. *Revue de Mycologie, Paris, Supplement Colonial*: 28
116. Mostert L, Crous PW, Petrini O. 2000. Endophytic fungi associated with shoots and leaves of Vitis vinifera, with specific reference to the Phomopsis viticola complex. *Sydowia* **52**: 46-58.
117. Murray DIL, Burpee LL. 1984. *Ceratobasidium cereale* sp.nov., the teleomorph of Rhizoctonia cerealis. *Transactions of the British Mycological Society* **82**: 170–172.
118. Mwenje E, Mguni N. 2001. Cellulolytic and pectinolytic activities of *Capnodium isolates* (sooty mould) from Zimbabwe. *Canadian Journal of Botany* **79**: 1492-1495.
119. Nakasone KK, Lindner DL. 2012. Taxonomy of *Pseudolagaro basidium* (Polyporales, Basidiomycota). *Fungal Diversity* **55**: 155-169.
120. Nazar RN, Hu X, Schmidt J, Culham D, Robb J. 1991. Potential use of PCR-amplified ribosomal intergenic sequences in the detection and differentiation of Verticillium wilt pathogens. *Physiological and Molecular Plant Pathology* **39**: 1-11.
121. Nelson PE, Wilhelm S. 1956. An Undescribed Fungus Causing a Root Rot of Strawberry. *Mycologia* **48**: 547-451.
122. Neuhaus JM, Ahl-Goy P, Hinz U, Flores S, Meins JrF. 1991. High-level expression of a tobacco chitinase gene in Nicotiana sylvestris. Susceptibility of transgenic plants to *Cercospora nicotianae* infection. *Plant molecular biology* **16**: 141-151.
123. Nierman WC, Pain A, Anderson MJ, Wortman JR, Kim HS, Arroyo J, Berriman M, Abe K, Archer DB, Bermejo C, et al. 2005. Genomic sequence of the pathogenic and allergenic filamentous fungus *Aspergillus fumigatus*. *Nature* **438**: 1151-1156.
124. Nishijima KA, Follett PA, Bushe BC, Nagao MA. 2002. First report of *Lasmenia sp.* and two species of Gliocephalotrichum on rambutan in Hawaii. *Plant Disease* **86**: 71-71.
125. Nishimura S, Sugihara M, Kohmoto K, Otani H. 1978. Two different phases in pathogenicity of the Alternaria pathogen causing black spot disease of Japanese pear. *Journal of the Faculty of Agriculture, Tottori University* **13**: 1-10.
126. Nita M, Ellis MA, Wilson LL, Madden LV. 2006. Evaluation of a disease warning system for Phomopsis cane and leaf spot of grape: a field study. *Plant disease***90**: 1239-1246.
127. Novotný Č, Erbanová P, Cajthaml T, Rothschild N, Dosoretz C, Šašek V. 2000. Irpex lacteus, a white rot fungus applicable to water and soil bioremediation. *Applied Microbiology and Biotechnology* **54**: 850-853.
128. Petersen D, Cation D. 1950. Exploratory experiments on the use of actidione for the control of *Peach brown* rot and Cherry leaf spot. *Plant Disease Reporter* **34**: 5-6.
129. Phillips DH, Burdekin DA. 1992. *Diseases of forest and ornamental trees*. Macmillan.
130. Prasher IB, Ashok D. 2013. A Checklist of Wood Rotting Fungi (non-gilled Agaricomycotina) of *Himachal Pradesh*. *Journal on New Biological Reports*, **3**: 71.
131. Qing F, Tian S. 2000. Postharvest biological control of Rhizopus rot of nectarine fruits by Pichia membranefaciens." Plant Disease 84, no. 11 (2000): 1212-1216.
132. Rahman M, Punja ZK. 2005. Factors influencing development of root rot on ginseng caused by *Cylindrocarpon destructans*. *Phytopathology*, **95**: 1381-1390.
133. Ramonell K, Berrocal-Lobo M, Koh S, Wan J, Edwards H, Stacey G, Somerville S. 2005. Loss-of-function mutations in chitin responsive genes show increased susceptibility to the powdery mildew pathogen Erysiphe cichoracearum. *Plant physiology* **138**: 1027-1036.
134. Reeleder RD. 2007. *Rhexocercosporidium panacis* sp. nov., a new anamorphic species causing rusted root of ginseng (*Panax quinquefolius*). *Mycologia* **99**: 91-98.
135. Reeleder RD, Hoke SMT, Zhang Y. 2006. Rusted root of ginseng (*Panax quinquefolius*) is caused by a species of Rhexocercosporidium. *Phytopathology* **96**: 1243-1254.
136. Reynolds DR. 1999. *Capnodium citri*: The sooty mold fungi comprising the taxon concept. *Mycopathologia* **148**: 141-147.
137. Rizzo DM, Harrington TC. 1988. Root and butt rot fungi on balsam fir and red spruce in the White Mountains, New Hampshire. *Plant Disease* **72**: 329-331.
138. Rodriguez RJ, Yoder OC. 1987. Selectable genes for transformation of the fungal plant pathogen *Glomerella cingulata* f. sp. Phaseoli (*Colletotrichum* *lindemuthianum*). *Gene* **54**: 73-81.
139. Rogers JD. 1984. *Xylaria acuta*, *Xylaria cornu-damae*, and *Xylaria mali* in continental United States. *Mycologia* **76**: 23-33.
140. Roy BA, Vogler DR, Bruns TD, Szaro TM. 1998. Cryptic species in the Puccinia monoica complex. *Mycologia* **90**: 846-853.
141. Royse DJ, Ries SM. 1978. The influence of fungi isolated from peach twigs on the pathogenicity of *Cytospora cincta*. *Phytopathology* **68**: 603.
142. Rozsnyay ZD. 1977. *Cytospora canker* and dieback of apricots. *EPPO Bulletin* **7**: 69-80.
143. Sánchez-Torres P, Hinarejos R, Tuset JJ. 2009. Characterization and pathogenicity of *Fusicladium eriobotryae*, the fungal pathogen responsible for loquat scab. *Plant Disease* **93**: 1151-1157.
144. Sankaran KV, Sutton BC, Balasundaran M. 1995. *Cryptosporiopsis* *eucalypti* sp. nov., causing leaf spots of eucalypts in Australia, India and USA. *Mycological Research* **99**: 827-830.
145. Santos JM, Vrandečić K, Ćosić J, Duvnjak T, Phillips AJL. 2011. Resolving the Diaporthe species occurring on soybean in Croatia. *Persoonia: Molecular Phylogeny and Evolution of Fungi*27: 9.
146. Saroj A, Kumar A, Qamar N, Alam M, Singh HN, Khaliq A. 2012. First report of wet rot of *Withania somnifera* caused by *Choanephora cucurbitarum* in India. *Plant Disease* **96**: 293.2.
147. Schroeder HW, Christensen JJ. 1963. Factors affecting resistance of wheat to scab caused by Gibberella zeae. *Phytopathology* **53**: 831-838.
148. Shearer JF. 1996. Potential of a pathogen, Mycoleptodiscus terrestris, as a biocontrol agent for the management of Myriophyllum spicatum in Lake Guntersville Reservoir. No. WES/TR/A-96-4. U.S. Army Engineer Waterways Experiment Station Vicksburg Ms Environmental Lab.
149. Shimanuki T. 1987. Studies on the mechanisms of the infection of timothy with purple spot disease caused by *Cladosporium phlei* (Gregory) de Vries. *Research Bulletin of the Hokkaido National Agricultural Experiment Station* **148**: 1-56.
150. Scattolin L, Montecchio L. 2007. First report of damping-off of common oak plantlets caused by *Cylindrocladiella parva* in Italy. *Plant Disease* **91**: 771-771.
151. Schmidhalter DR, Canevascini G. 1993. Isolation and Characterization of the Cellobiose Dehydrogenase from the Brown-Rot Fungus *Coniophora puteana* (Schum ex Fr.) Karst. *Archives of Biochemistry and Biophysics***300**: 559-563.
152. Shimizu T, Nakatsu T, Miyairi K, Okuno T, Kato H. 2001. Crystallization and preliminary X-ray study of endopolygalacturonase from the pathogenic fungus Stereum purpureum. *Acta Crystallographica Section D: Biological Crystallography* **57**: 1171-1173.
153. Shimizu T, Shibata H, Araya T, Nakatsu T, Miyairi K, Okuno T, Kato H. 2005. Expression, purification, and crystallization of endopolygalacturonase from a pathogenic fungus, *Stereum purpureum*, in *Escherichia coli*. *Protein Expression and Purification* **44**: 130-135.
154. Siddiqui Y, Meon S, Ismail R, Rahmani M. 2009. Bio-potential of compost tea from agro-waste to suppress *Choanephora cucurbitarum* L. the causal pathogen of wet rot of okra. *Biological Control* **49**: 38-44.
155. Smith BJ, Black LL. 1990. Morphological, cultural and pathogenic variation among Colletotrichum species isolated from strawberry. *Plant Disease* **74**: 69-76.
156. Snyder WC, Hansen HN. 1940. The species concept in Fusarium. American *Journal of Botany* **27**: 64-67.
157. Soares DJ, Fabiano BR, Lidiane LD, Barreto RW. 2011. *Pyriculariopsis calatheae* sp. nov., a novel anamorphic hyphomycete from the Atlantic forest of Brazil causing leaf spots on *Calathea longifolia*. *Mycological Progress***10**: 315-321.
158. Graham J.H. 1951. Diseases of cereals and grasses in North America. *Agronomy Journal* **43**: 152.
159. Stalpers JA, Loerakker WM. 1982. Laetisaria and Limonomyces species (Corticiaceae) causing pink diseases in turf grasses. *Canadian Journal of Botany*, **60**: 529-537.
160. Stavishenko IV, Zalesov SV, Luganskii NA, Kryazhevskikh NA, Morozov AE. 2002. Communities of wood-attacking fungi in the region of oil and gas production. *Russian Journal of Ecology* **33**: 161-169.
161. Stukenbrock EH, Jørgensen FG, Zala M, Hansen TT, McDonald BA, Schierup MH. 2010. Whole-genome and chromosome evolution associated with host adaptation and speciation of the wheat pathogen Mycosphaerella graminicola. *PLoS Genetics* **6**: e1001189.
162. Sutton JC. 1982. Epidemiology of wheat head blight and maize ear rot caused by *Fusarium graminearum*. *Canadian Journal of Plant Pathology* **4**: 195-209.
163. Swart L, Crous PW, Denman S, Palm ME. 1998. Fungi occurring on proteaceae. I. *South African Journal of Botany* **64**: 137-145.
164. Talibi I, Askarne L, Boubaker H, Boudyach EH, Msanda F, Saadi B, Ait Ben Aoumar A. 2012. Antifungal activity of some Moroccan plants against *Geotrichum candidum*, the causal agent of postharvest citrus sour rot. *Crop Protection* **35**: 41-46.
165. Tanaka E, Tanaka C, Shibata S. 2009. Bamboo witches' broom in Japan. *Transactions of the Mycological Society of Japan* **50**: 56-60.
166. Taylor JE, Crous PW, Wingfield MJ. 1999. *Batcheloromyces* species occurring on Proteaceae in South Africa. *Mycological Research* **103**: 1478-1484.
167. Thal WM, Campbell CL. 1986. Spatial pattern analysis of disease severity data for alfalfa leaf spot caused primarily by *Leptosphaerulina briosiana*. *Phytopathology* **76**: 190-194.
168. Thomashow LS, Weller DM. 1988. Role of a phenazine antibiotic from Pseudomonas fluorescens in biological control of Gaeumannomyces graminis var. tritici. *Journal of Bacteriology* **170**: 3499-3508.
169. Timmermann V, Børja I, Hietala AM, Kirisits T, Solheim H. 2011. Ash dieback: pathogen spread and diurnal patterns of ascospore dispersal, with special emphasis on Norway*. *EPPO Bulletin* **41**: 14-20.
170. Tisserat N, Cranshaw W, Putnam ML, Pscheidt J, Leslie CA, Murray M, Hoffman J, Barkley Y, Alexander K, Seybold SJ. 2011. Thousand cankers disease is widespread in black walnut in the western United States. *Plant Health Progress* doi:10.1094/PHP-2011-0630-01-BR.
171. Tzeng TH, Lyngholm LK, Ford CF, Bronson CR. 1992. A restriction fragment length polymorphism map and electrophoretic karyotype of the fungal maize pathogen Cochliobolus heterostrophus. *Genetics* **130**: 81-96.
172. Tushemereirwe WK, Waller JM. 1993. Black leaf streak (*Mycosphaerella fijiensis*) in Uganda. *Plant Pathology*, **42**: 471-472.
173. Unestam T, Beyer-Ericson L, Strand M. 1989. Involvement of *Cylindrocarpon destructans* in root death of Pinus sylvestris seedlings: pathogenic behaviour and predisposing factors. *Scandinavian Journal of Forest Research* **4**: 521-535.
174. Vakili NG. 1985. Mycoparasitic fungi associated with potential stalk rot pathogens of corn. *Phytopathology* **75**: 1201-1207.
175. Wang X, Wei J, Huang L, Kang Z. 2011. Re-evaluation of pathogens causing Valsa canker on apple in China. *Mycologia* **103**: 317-324.
176. Watanabe T, Moya JDD, González JL, Matsuda A. 1997. Mycoleptodiscus terrestris from black pepper roots in the Dominican Republic. *Mycoscience* **38**: 91-94.
177. Wei CT. 1950. Notes on Corynespora. *Mycological Papers* **34**: 10.
178. Weiss F. 1940. Anthracnose and Cladosporium stem spot of Peony. *Phytopathology* **30**: 409-417.
179. Welty RE, Lucas GB, Fletcher JT, Yang H. 1968. Fungi isolated from tobacco leaves and brown-spot lesions before and after flue-curing. *Applied microbiology* **16**: 1309-1313.
180. West JS, Kharbanda PD, Barbetti MJ, Fitt BDL. 2001. Epidemiology and management of *Leptosphaeria maculans* (phoma stem canker) on oilseed rape in Australia, Canada and Europe. *Plant pathology* **50**: 10-27.
181. Wilson CL, Franklin JD, Pusey PL. 1987. Biological control of Rhizopus rot of peach with Enterobacter cloacae. *Phytopathology* **77**: 303-305.
182. Winton LM, Stone JK, Hansen EM, Shoemaker RA. 2007. The systematic position of Phaeocryptopus gaeumannii. *Mycologia* **99**: 240-252.
183. Wu J, Zheng F. 2011. Pathogen identification and fungicide screening of the leaf blight of strawberry. *Plant Protection* **6**: 036.
184. Zhang M, Wu HY, Tsukiboshi T, Okabe I. 2010. First report of *Pestalotiopsis microspora* causing leaf spot of Hidcote (*Hypericum patulum*) in Japan. *Plant Disease* **94**: 1064-1064.
